# ﻿Four new species of *Ditrigona* Moore (Lepidoptera, Drepanidae) in China and an annotated catalogue

**DOI:** 10.3897/zookeys.1091.78986

**Published:** 2022-03-31

**Authors:** Xiao-Jiang Guo, Rui Cheng, Shan Jiang, Da-Yong Xue, Hong-Xiang Han

**Affiliations:** 1 Key Laboratory of Zoological Systematics and Evolution, Institute of Zoology, Chinese Academy of Sciences, Beijing 100101, China Institute of Zoology, Chinese Academy of Sciences Beijing China; 2 College of Life Science, Hebei University, Baoding, Hebei 071000, China Hebei University Baoding China

**Keywords:** DNA barcoding, Drepaninae, new combination, taxonomy

## Abstract

The Chinese species of the genus *Ditrigona* Moore, 1888 are reviewed and an annotated catalogue is provided. Four new species are described from China: *Ditrigonasinespina* Jiang & Han, **sp. nov.**, *Ditrigonaparva* Jiang & Han, **sp. nov.**, *Ditrigonaconcava* Guo & Han, **sp. nov.**, and *Ditrigonafusca* Guo & Han, **sp. nov.***Derocacrystalla* Chu & Wang, 1987 and *Auzatellapentesticha* Chu & Wang, 1987 are newly combined into, respectively, the *derocina* and *quinaria* species groups of *Ditrigona*. *Ditrigonadiana* Wilkinson is newly recorded in China. This results in 43 species of *Ditrigona* for the fauna of China. Illustrations of habitus and genitalia of the new species and most known species are presented.

## ﻿Introduction

The genus *Ditrigona* was originally established by Moore (1888) on the basis of *Urapteryxtriangularia* Moore, 1868 from India. Later, Warren (1922) described *Ditrigonaregularis* Warren from Assam, and Bryk (1943) described *Ditrigonaregularisdifferentiata* Bryk from Burma. After a long silence in research into *Ditrigona*, Wilkinson (1968) provided the most comprehensive revision of the genus. He established three new generic synonyms of the genus, and transferred most of the species involved into *Ditrigona*; altogether he recorded 40 species and 12 subspecies for the genus, including the description of 18 new species and five new subspecies. He also placed the species into four species groups, and provided descriptions and diagnosis of the genus, species groups, species, and subspecies. More recently, on the basis of Wilkinson’s work, Chu and Wang (1988) recorded 36 species from China, including description of a new species *Ditrigonauniuncusa* Chu & Wang, and these species were included in vol. 3 of *Fauna Sinica* (Chu and Wang 1991). Holloway (1998) described two species *Ditrigonapaludicola* and *Ditrigonawilkinsoni* from Borneo. The most recent research was provided by Li et al. (2015), who described *Ditrigonaclavata* from Guangdong, China, and by Jiang and Han (2019), who described *Ditrigonatenuiata* from Sichuan, China and provided a checklist of the *triangularia* species group.

Further study of the specimens of *Ditrigona* from IZCAS and MHBU shows that four new species need to be described. The purposes of this paper are to provide a survey and an annotated catalogue of Chinese *Ditrigona* species, to describe four new species, to newly record *Ditrigonadiana* Wilkinson, 1968 from China, to transfer two species into the genus, and to provide illustrations of external features and genitalia of new species and most known species. This results in 43 species and 8 subspecies of *Ditrigona* for the fauna of China.

## ﻿Materials and methods

The depositories of all the types and examined specimens are indicated as follows:

**NHMUK**The Natural History Museum, London, UK;

**ZFMK**Zoologisches Forschungsmuseum Alexander Koenig, Bonn, Germany;

**NHRS**Naturhistoriska Riksmuseet, Stockholm, Sweden;

**IZCAS**Institute of Zoology, Chinese Academy of Sciences, Beijing, China;

**MHBU** The Museum of Hebei University, Baoding, China;

**XTBG** Xishuangbanna Tropical Botanical Garden, Chinese Academy of Sciences, Yunnan, China;

**SCAU** South China Agricultural University, Guangzhou, China;

**MNHN**Muséum National d’Histoire Naturelle, Paris, France;

**DEI**Deutsches Entomologisches Institut, Germany.

Terminology for the genitalia is based on Wilkinson (1968). Moths were photographed with a digital camera (Canon Pc1057). Composite images were generated using Auto-Montage software version 5.03.0061 (Synoptics Ltd). The sharpness-contrast of the photos was enhanced and the plates compiled using Adobe Photoshop (CS 5.1).

A total of 16 specimens of the species of the *triangularia* species group bearing a tail process were used for sequencing the DNA barcoding region of the mitochondrial COI gene. DNA barcodes of 15 specimens were successfully obtained in this work, and one sequence of *D.concava* was downloaded from BOLD: its related voucher specimen was donated by Prof. Akihior Nakamura from Xishuangbanna Tropical Botanical Garden, Chinese Academy of Sciences (XTBG). Four of these specimens were *D.regularis*, one of *D.triangularia*, two *D.tenuiata*, four *D.sinespina* sp. nov., three *D.parva* sp. nov., and two *D.concava* sp. nov.

Protocols of DNA extraction and sequencing followed Ban et al. (2018). Details of studied specimens, including GenBank and BOLD accession numbers are summarized in Table 1. Pairwise distances within and between *Ditrigona* species for the COI barcoding region (612 bp) were calculated, and a neighbour-joining (NJ) tree (Saitou and Nei 1987) was constructed based on the Kimura two-parameter (K2P) method (Kimura 1980) using MEGA 6.0.

**Table 1.** Details of specimens used in molecular analysis of the DNA barcode region.

**Sample ID**	**Species**	**Date Collected**	**Locality**	**Collectors**	**GenBank/BOLD accession number**
LEP M 33040	*D.parva*	6–8.Aug.2016	Tengchong, Yunnan	Ban XS	OL664050
LEP M 33049	*D.parva*	6–8.Aug.2016	Tengchong, Yunnan	Ban XS	OL664048
LEP M 33059	*D.parva*	6–8.Aug.2016	Tengchong, Yunnan	Ban XS	OL664049
LEP M 33016	*D.regularis*	9–12.Aug.2016	Yunlong, Yunnan	Ban XS	MK087682
LEP M 33027	*D.regularis*	6–8.Aug.2016	Tengchong, Yunnan	Ban XS	MK087683
LEP M 32911	*D.regularis*	10–13.Aug.2017	Xinping, Yunnan	Cui L	MK087678
LEP M 35671	*D.regularis*	14–16.Jul.2018	Anha, Sichuan	Cui L, Jiang S	MK087688
LEP M 32976	*D.triangularia*	13–14.Jul.2014	Weixi, Yunnan	Pan XD	MK087679
LEP M 25081	*D.tenuiata*	11.Sep.2016	Luding, Sichuan	Li XX	MK087687
LEP M 23038	*D.tenuiata*	7–10.Aug.2016	Kangding, Sichuan	Cui L	MK087685
LEP M 33029	*D.sinespina*	9–12.Aug.2016	Yunlong, Yunnan	Ban XS	MK087684
LEP M 33001	*D.sinespina*	9–12.Aug.2016	Yunlong, Yunnan	Ban XS	MK087680
LEP M 33002	*D.sinespina*	9–12.Aug.2016	Yunlong, Yunnan	Ban XS	MK087681
LEP M 33196	*D.sinespina*	26–27.Jun.2014	Tengchong, Yunnan	Pan XD	MK087677
LEP M 32975	*D.concava*	13–14.Jul.2014	Weixi, Yunnan	Li XX	OL664047
ARB00027811	*D.concava*	11.Aug.2011	Ailao Shan, Yunnan	Kitching RL, Ashton LA	SCDBC000200

## ﻿Systematics

### ﻿Genus Ditrigona

Taxon classificationAnimaliaLepidopteraDrepanidae

Moore, 1888

2F199891-6CC1-557F-9D29-E50F4AD4D8E0

Ditrigona Moore, 1888: 258. Type species: Urapteryxtriangularia Moore, 1867.
Leucodrepana Hampson, 1893: 333. Type species: Leucodrepanaidaeoides Hampson, 1892.
Leucodrepanilla Strand, 1911: 198. Type species: Coryciasacra Butler, 1878.
Auzatella Strand, 1917: 148. Type species: Auzatamicronioides Strand, 1917.
Thaleridia Moore, 1888: 266. Type species: Thaleridiapruinosa Moore, 1888.

#### Generic characters.

The generic characters of *Ditrigona* and its differentiation from related genera are detailed in Wilkinson (1968) and Jiang and Han (2019).

#### Distribution.

The species of *Ditrigona* are mainly distributed in the Oriental region.

##### *derocina* species group

Wilkinson (1968) placed three species in the *derocina* species group: *Ditrigonaderocina* (Bryk), *Ditrigonadiana* Wilkinson, and *Ditrigonapruinosa* (Moore). The two former species have been recorded in China, and a further species, *Derocacrystalla* Chu & Wang, 1987 is newly placed in this species group in this work.

Species of the *derocina* species group are characterized by unipectinate antennae and semi-transparent wings. In the male genitalia, the *derocina* species group is unusual in *Ditrigona* in having sclerotization of the vinculum, and a quite long and narrow aedeagus. The eighth sternite is distinguished by large and curved octavals, and the tergite protrudes strongly. The female genitalia are characterized by having a very long ductus bursae, an ostial plate, an accessory sac and a long and narrow signum.

### ﻿1. Ditrigona derocina

Taxon classificationAnimaliaLepidopteraDrepanidae

(Bryk, 1943)

E36D0FA1-5CDD-57A5-885B-31273B134600

**Figures 1–27.**
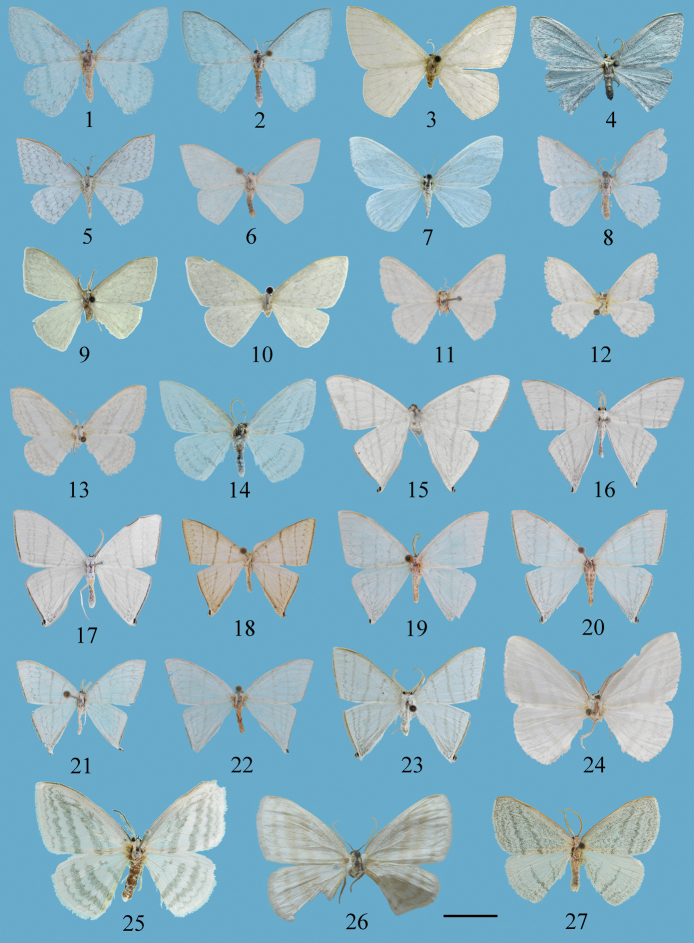
Adults of *Ditrigona***1***D.derocina*, male **2***D.diana*, male **3***D.crystalla*, holotype, male **4***D.spilota*, male, ZFMK**5***D.furvicosta*, male **6***D.jardanaria*, male **7***D.media*, paratype, male, ZFMK**8***D.sericea*, male **9***D.sericea*, male (*Auzatellapentesticha* Chu & Wang, allotype) **10***D.pentesticha*, holotype, female **11***D.q.erminea*, holotype, male, ZFMK**12***D.q.spodia*, holotype, male, ZFMK**13***D.q.leucophaea*, holotype, male, ZFMK**14***D.obliquilineathibetaria*, male **15***D.triangularia*, lectotype, male **16***D.uniuncusa*, male **17***D.tenuiata*, holotype, male **18***D.regularis*, male **19, 20***D.sinespina* sp. nov., **19** holotype, male **20** paratype, female **21, 22***D.parva* sp. nov., **21** holotype, male **22** paratype, female **23***D.concava* sp. nov., holotype, male **24***D.titana*, holotype, male, ZFMK**25***D.pomenaria* male **26***D.polyobotaria*, holotype, female, ZFMK**27***D.typhodes*, male. Scale bar: 1 cm.

Figs 1 , 48 , 82 , 116 , 152

Peridrepana derocina Bryk, 1943: 6. Holotype ♀, Burma: Kambaiti (NHRS).
Ditrigona derocina : Wilkinson, 1968: 418.

#### Material examined.

**China: Hubei** (IZCAS): 1♂7♀, Xuanen, Liangxihe, 796 m, 20–22.IX.2015, leg. Yao Jian, Zhao Kaidong. **Hunan** (IZCAS): 2♀, Sangzhi, Badagong Shan, Xiaozhuangping, 1420 m, 14.VI.2015, leg. Yao Jian, Zhao Kaidong. **Sichuan** (IZCAS): 1♀, Emei Shan, 0km, 1288 m, 31.VII.2013, leg. Cheng Rui. **Chongqing** (IZCAS): 1♂, Wu Shan, Wulipo, Dangyangcongping, 1773 m, 25.VII.2013, leg. Cheng Rui. **Yunnan** (IZCAS): 1♀, Lushui, Yaojiaping, 2500 m, 4.VI.1981, leg. Liao Subai; 2♂1♀, Tengchong, Houqiao, 1620 m, 6–8.VIII.2016, leg. Ban Xiaoshuang; 3♀, Tengchong, Heinitang, 1930 m, 28–30.V.1992, leg. Xue Dayong; 1♂1♀, same locality, 1824 m, 26–27.VI.2014, leg. Li Xinxin, Pan Xiaodan; 2♂3♀, Tengchong, Dahaoping, 2020 m, 24–26.V.1992, leg. Xue Dayong; 5♂2♀, same locality, 2020 m, 5–7.VIII.2007, leg. Wu Chunguang, Xue Dayong; 3♀, Lushui, Pianma, 1980 m, 3–4.VII.2014, leg. Pan Xiaodan; 1♀, Pianma, Dianxin hotel, 1970 m, 8–12.V.2011, leg. Yang Xiushuai, Wang Ke; 1♀, Gongshan, Puladi, 1298 m, 6–7.VII.2014, leg. Pan Xiaodan. **Tibet** (IZCAS): 1♂, Zham, 2400 m, 4.VII.1975, leg. Wang Ziqing; 2♀, same locality and collector, 2200 m, 23–30.VI.1957; 1♂1♀, Bomi, Tangmai, 2000 m, 26–28.VI.2015, leg. Li Xinxin; 1♀, Bomi, Tangmaidaqiao, 2037 m, 13–14.VI.2016, leg. Li Xinxin. **India** (ZFMK): 1♂, Sikkim, Namchi, 1000 m, 2.VIII.1986, leg. W. Thomas, photograph examined.

#### Distribution.

China (Hubei, Hunan, Sichuan, Chongqing, Yunnan, Tibet), India, Myanmar.

### ﻿2. Ditrigona diana

Taxon classificationAnimaliaLepidopteraDrepanidae

Wilkinson, 1968; new record for China

26A7B8F1-E4F4-5749-983A-2564E64D7D44

Figs 2 , 49 , 83 , 117 , 153

Ditrigona diana Wilkinson, 1968: 420. Holotype ♂, India: Gopaldara (NHMUK).

#### Material examined.

**India**: 1♂ (ZFMK), paratype, Khasis, IV.1895, Nat. Coll., Collectio. H.J. Elwes, moth photographed examined. **China: Guangxi** (IZCAS): 1♂1♀, Napo, Defu, 1350 m, 19.VI.2000, leg. Li Wenzhu. **Yunnan** (IZCAS): 1♀, Xishuangbanna, Mengla, Menglun, 550 m, 12–15.V.2017, leg. Jiang Shan; 5♂2♀, Xishuangbanna, Mengla, Bubang, 680 m, 18–20.V.2017, leg. Jiang Shan; 1♂, Tengchong, Zhengding, 1833 m, 6–7.VIII.2013, leg. Li Xinxin; 1♂, Pingbian, Daweishan, 2090 m, 4–8.VIII.2017, leg. Cui Le; 1♂, Baoshan, Baihualing, 1520 m, 11–13.VIII.2007, leg. Wu Chunguang; 1♀, Tengchong, Dahaoping, 2020 m, 5–7.VIII.2007, leg. Wu Chunguang; 1♀, Tengchong, Heinitang, 1824 m, 26–27.VI.2014, leg. Pan Xiaodan; 1♀, Ruili, Wanding, Forest Garden, 900 m, 29.IV.2011, leg. Yang Xiushuai, Wang Ke; 2♀, Ruili, Wanding, Tianehu, 923 m, 30.IV.–1.V.2011, leg. Yang Xiushuai, Wang Ke; 8♀, Ruili, Mengmao, Mangling, 900 m, 26–27.IV.2011, leg. Yang Xiushuai, Wang Ke. **Tibet** (IZCAS): 1♂2♀, Mêdog, Yarang, 1091 m, 20–23.VIII.2006, leg. Lang Songyun.

#### Distribution.

China (Guangxi, Yunnan, Tibet), India.

### ﻿3. Ditrigona crystalla

Taxon classificationAnimaliaLepidopteraDrepanidae

(Chu & Wang, 1987) comb. nov.

7CD61358-E898-5F44-B58D-82698560BB05

Figs 3 , 50 , 84 , 118 , 154

Deroca crystalla Chu & Wang, 1987: 116. Holotype ♂, China: Yunnan: Lushui: Yaojiaping (IZCAS).

#### Note.

The species *Derocacrystalla* Chu & Wang, 1987 was described from Yunnan, Sichuan and Tibet. Its male genitalia obviously belong to the *derocina* species group of *Ditrigona*, and we therefore transfer the species to *Ditrigona*. The male genitalia are almost identical to those of *D.derocina*. However, the corpus bursae of the female genitalia is scobinate, which is different from the smooth ones of *D.derocina* and *D.diana*, though they share a very long and narrow ductus bursae and a slender curved signum. The ostial plate is invisible in *D.crystalla*, and it is most probably present, though it seems that the sternite was incorrectly removed and the abdomen is not preserved on the slide.

#### Material examined.

**China: Yunnan** (IZCAS): 1♂, holotype of *Derocacrystalla*, Lushui, Yaojiaping, 2500 m, 4.VI.1981, leg. Liao Subai; 1♂, Tengchong, Dahaoping, 2020 m, 24–26.V.1992, leg. Xue Dayong; 1♂, same locality, 5–7.VIII.2007, leg. Wu Chunguang, Xue Dayong. **Sichuan** (IZCAS): 1♀, allotype of *Derocacrystalla*, Guan Xian, Qingcheng Shan, 700–1600 m, 4.VI.1979, leg. Shang Jinwen. **Tibet** (IZCAS): 1♂, paratype of *Derocacrystalla*, Zham, 2400 m, 4.VII.1975, leg. Wang Ziqing.

#### Distribution.

China (Sichuan, Yunnan, Tibet).

##### *quinaria* species group

Wilkinson (1968) recorded 11 species in the *quinaria* species group: *Ditrigonaspilota* Wilkinson, *Ditrigonainconspicua* (Leech), *Ditrigonafurvicosta* (Hampson), *Ditrigonajardanaria* (Oberthür), *Ditrigonamedia* Wilkinson, *Ditrigonainnotata* (Hampson), *Ditrigonasericea* (Leech), *Ditrigonaquinaria* (Moore), *Ditrigonaobliquilinea* (Hampson), *Ditrigonaidaeoides* (Hampson), and *Ditrigonaspatulata* Wilkinson. The former 10 species are recorded in China, and *Auzatellapentesticha* Chu & Wang is newly combined to *Ditrigona* in this work.

Species of the *quinaria* species group share bipectinate or serrate antennae with some species of the *triangularia* and *mytylata* species groups. In the male genitalia, the uncus is usually single, but sometimes bifurcate or notched. The single uncus resembles that of species of the *derocina* species group, but the group can be differentiated by the lack of sclerotization on the vinculum, and the large and broad saccus. The small and setose valva lacking a posterior projection also differs from those in the *triangularia* and *mytylata* species groups. The aedeagus is often characterized by the presence of a minute to large apical projection (not present in *D.spilota* and *D.obliquilinea*, and the situation unknown in *D.innotata* and *D.idaeoides*). The eighth sternite is modified with short octavals, and the tergite is often shallowly to moderately concave, occasionally straight or protruding with tiny lateral projections. The female genitalia lack an ostial plate; the ductus bursae is short and broad, and the corpus bursae usually has an accessory sac and a long and thin signum.

### ﻿4. Ditrigona spilota

Taxon classificationAnimaliaLepidopteraDrepanidae

Wilkinson, 1968

BFF16FDA-163C-5E18-8100-A5BFF891C46B

Figs 4 , 51 , 85 , 119 , 155

Ditrigona spilota Wilkinson, 1968: 423. Holotype ♂, China: Yunnan, Likiang (ZFMK).

#### Material examined.

**China: Yunnan**: 1♂ (ZFMK), paratype, Li-kiang (China), Provinz Nord-Yuennan, 16.VIII.1935, H. Höne, dissected in this work; 1♀ (ZFMK), same locality, 20.VIII.1935, H. Höne, dissected in this work; 1♀ (IZCAS), Tengchong, Danzhalinchang, 2500 m, 2–4.VI.1992, leg. Xue Dayong; 1♂1♀ (IZCAS), Lijiang, 3700 m, 9.VIII.2012, leg. Ashton; 2♂, same locality and collector, 3400 m, 17.VIII.2012. **Sichuan** (IZCAS): 1♀, Luding, Moxi, Hailuogou, 2596 m, 12.IX.2016, leg. Li Xinxin; 1♀, Luding, Moxi, Hailuogou Guancezhan, 3000 m, 10.IX.2016, leg. Li Xinxin.

#### Distribution.

China (Sichuan, Yunnan).

#### Remarks.

Compared to the male genitalia of the holotype (fig. 17 in Wilkinson 1968) of *D.spilota*, the socii of the paratype examined are much broader and blunter, while the aedeagus and 8^th^ segment have no distinct differences. Further study is needed to investigate whether this is intraspecific variation, or more than one species is present in the large type series.

### ﻿5. Ditrigona inconspicua

Taxon classificationAnimaliaLepidopteraDrepanidae

(Leech, 1898)

5BADEC1D-6FE0-5C10-9386-03B6C7272307

Teldenia inconspicua Leech, 1898: 363. Lectotype ♂, China: Sichuan, Ta-Chien-lu (NHMUK).
Peridrepana inconspicua : Warren, 1922: 449.
Ditrigona inconspicua : Wilkinson, 1968: 425.

#### Material examined.

No.

#### Distribution.

China (Sichuan).

### ﻿6. Ditrigona furvicosta

Taxon classificationAnimaliaLepidopteraDrepanidae

(Hampson, 1912)

2BA06E73-207B-5A30-9A09-46475734705F

Figs 5 , 52 , 86 , 120 , 156

Leucodrepana furvicosta Hampson, 1912: 1271. Lectotype ♂, India: Sikkim (NHMUK).
Ditrigona furvicosta : Wilkinson, 1968: 428.

#### Material examined.

**China: Yunnan**: 1♀ (IZCAS), Tengchong, Danzhalinchang, 2500 m, 2–4.VI.1992, leg. Xue Dayong; 1♂1♀ (IZCAS), same locality, 2479 m, 30.VI.–1.VII.2014, leg. Pan Xiaodan; 2♂ (IZCAS), Lijiang, Alpine Botanical Garden, 3260–3452 m, 20.VI.2009, leg. Qi Feng; 1♂ (ZFMK), Li-kiang (China), Provinz Nord-Yuennan, 23.VI.1935, H. Höne, moth photograph examined. **Tibet** (IZCAS): 2♀, Yadong, Yadonglinchang, 2690 m, 24.VI.2016, leg. Li Xinxin; 1♂3♀, Nyingchi, Pêlung, 2115 m, 1.IX.2005, leg. Wang Xuejian; 1♂ (MHBU), Zham, 27.VII.2005, leg. Shi Aimin.

#### Distribution.

China (Yunnan, Tibet), India.

### ﻿7. Ditrigona jardanaria

Taxon classificationAnimaliaLepidopteraDrepanidae

(Oberthür, 1923)

8720AF45-59A3-5576-8F21-3FC66FE17C1B

Figs 6 , 53 , 87 , 121

Corycia jardanaria Oberthür, 1923: 238. Lectotype ♂, China: Sichuan, Ta-tsien-lu (ZFMK).
Ditrigona jardanaria : Wilkinson, 1968: 429.

#### Material examined.

**China: Henan** (IZCAS): 1♂, Baiyun Shan, 1550 m, 13–15.VIII.2008, leg. Xue Dayong, Song Wenhui. **Shaanxi** (IZCAS): 1♂, Ningshan, Huoditang, 1520 m, 13–17.VIII.2016, leg. Cheng Rui, Jiang Shan. **Sichuan**: 1♂ (IZCAS), Luding, Hailuogou, 3010 m, 2–4.VIII.2014, leg. Pan Xiaodan; 1♂ (ZFMK), Ta-tsien-Lou, 1899, Chasseurs indigenes, Ex. Oberthür Coll., Brit. Mus. 1927-3, moth photograph examined.

#### Distribution.

China (Henan, Shaanxi, Sichuan, Tibet).

### ﻿8. Ditrigona media

Taxon classificationAnimaliaLepidopteraDrepanidae

Wilkinson, 1968

C9D22C52-FC1F-5223-A37C-3E64DD6A12AF

Figs 7 , 54 , 88 , 122

Ditrigona media Wilkinson, 1968: 431. Holotype ♂, China: Sichuan, Ta-tsien-Lou (NHMUK).

#### Material examined.

**China: Sichuan** (ZFMK): 1♂, paratype, Batang, Tibet [Sichuan], Alpine Zone, ca. 5000 m, 7.VI.1936, H. Höne, moth photograph examined; 1♂, same locality, 23.VI.1938, H. Höne, dissected in this work. **Gansu** (IZCAS): 1♂, Dangchang, Guanegou, 2045 m, 1–3.VIII.2016, leg. Cheng Rui, Jiang Shan.

#### Distribution.

China (Gansu, Sichuan, Tibet).

### ﻿9. Ditrigona innotata

Taxon classificationAnimaliaLepidopteraDrepanidae

(Hampson, 1893)

5BBBE430-9C7E-559D-82F1-0007E9ACE5AF

Drepana innotata Hampson, 1893: 335. Lectotype ♀, China: Kulu (Young) (NHMUK).
Peridrepana innotata : Warren, 1922: 449.
Ditrigona innotata : Wilkinson, 1968: 433.

#### Material examined.

No.

#### Distribution.

China (Tibet).

### ﻿10. Ditrigona sericea

Taxon classificationAnimaliaLepidopteraDrepanidae

(Leech, 1898)

3507BDBD-4075-549B-B444-98BD27B1C9E7

Figs 8–9 , 55–56 , 89 , 90 , 123 , 157

Teldenia sericea Leech, 1898: 263. Lectotype ♂, China: Sichuan, Moupin (NHMUK).
Drepana fulvicosta Dudgeon, 1899: 652. Syntype, India.
Peridrepana fulvicosta : Gaede, 1931: 7.
Leucodrepana nivea brimanica Bryk, 1943: 7. Holotype ♀ (as ♂): Burma: Kambaiti (NHRS).
Ditrigona sericea : Wilkinson, 1968: 434.

#### Material examined.

**China: Shaanxi** (IZCAS): 2♀, Ningshan, Huoditang, 1520 m, 13–17.VIII.2016, leg. Cheng Rui, Jiang Shan. **Sichuan**: 3♂1♀ (IZCAS), Luding, Moxi, Boyangcun, 1691 m, 1.VIII.2014, leg. Li Xinxin; 1♀ (IZCAS), Baoxing, Dashuigou Guanhuzhan, 1591 m, 1–5.VIII.2016, leg. Cui Le; 1♀ (IZCAS), Emei Shan, Qingyinge, 800–1000 m, 14.V.1957, leg. Zhu Fuxing; 1♂ (ZFMK), Ta-Tsien-Lou, Tche To, Chasseurs Indigènes, 1894, Ex. Oberthür Coll., Brit. Mus. 1927-3, moth photograph examined; 1♂ (ZFMK), Siao-Lou, 1903, Coll. R. P. Déjean. **Yunnan** (IZCAS): 1♂1♀, Tengchong, Houqiao, 1553 m, 28–29.VI.2014, Pan Xiaodan, Li Xinxin; 11♂17♀, same locality, 1620 m, 6–8.VIII.2016, leg. Ban Xiaoshuang; 1♀, same locality, 1080 m, 31.V.–1.VI.1992, leg. Xue Dayong; 3♂5♀, Tengchong, Dahaoping, 2020 m, 24–26.V.1992, leg. Xue Dayong; 3♂1♀, same locality, 2020 m, 5–7.VIII.2007, leg. Xue Dayong; 5♂1♀, same locality and date, leg. Wu Chunguang; 1♂2♀, ibidem, leg. Lang Songyun; 1♂2♀, Tengchong, Heinitang, 1824 m, 26–27.VI.2014, leg. Pan Xiaodan; 1♀, same locality and date, leg. Li Xinxin; 1♂, Tengchong Shidi, 1730 m, 3–5.VIII.2016, leg. Ban Xiaoshuang; 1♂3♀, Tengchong, Qushi, Dabacun, 1873 m, 4.VIII.2013, leg. Liu Shuxian; 2♂, same locality, 1823 m, 5.VIII.2013, leg. Liu Shuxian; 15♂5♀, Gongshan, Dulongjiang, 1505 m, 8–9.VII.2014, leg. Pan Xiaodan; 1♀, Gaoligong, Nankang, 2000 m, 21.III.2007, leg. Zhang Peiyi; 1♀, Gaoligong, Baihualing, 1500 m, 16.IX.2007, leg. Zhang Peiyi; 1♂, Baoshan, Bawan, 1040 m, 8–10.VIII.2007, leg. Wu Chunguang; 1♂, Pingbian, Dawei Shan, 2043 m, 19–20.VII.2016, leg. Ban Xiaoshuang; 5♂7♀, same locality, 2090 m, 4–8.VIII.2017, leg. Cui Le; 1♀, Yongsheng, Liude, 2300 m, 9.VII.1984, leg. Chen Yixin; 3♂1♀, Kunming, Xishan, 2100 m, 23.III.1958, leg. Meng Xuwu; 1♂, Lushui, Pianma, 1980 m, 3–4.VII.2014, leg. Li Xinxin; 1♂, Weixi, Pantiange, 2570 m, 15–16.VII.2014, leg. Pan Xiaodan; 2♂, Dali, Cangshan, 2226 m, 23–24.VI.2014, leg. Pan Xiaodan; 3♂1♀, same locality and date, leg. Li Xinxin; 1♂1♀, Yunlong, Tianchi, 2570 m, 9–12.VIII.2016, leg. Ban Xiaoshuang; 1♂2♀, Yunlong, Tianchi, 2570 m, 9–12.VIII.2016, leg. Ban Xiaoshuang; 2♂, Ailao Shan, 2000 m, 19–20.VIII.2011, leg. Ashton, ex. XTBG. **Tibet** (IZCAS): 1♂, allotype of *Auzatellapentesticha* Chu & Wang, 1987, Zham, 2400 m, 26.VI.1975, leg. Huang Fusheng; 1♂, Bomi, Tangmai, 2000 m, 26–28.VI.2015, leg. Li Xinxin; 2♂7♀, Mêdog, Hanmi, 2095 m, 10–11.VIII.2006, leg. Lang Songyun; 1♂3♀, Mêdog, Aniqiao, 1060 m, 12–13.VIII.2006, leg. Lang Songyun; 1♀, Mêdog, Dayandong, 2880 m, 9.VIII.2006, leg. Lang Songyun; 1♀, Mêdog 108K, 848 m, 4.VIII.2014, leg. Cheng Rui, Cui Le; 1♂2♀, Nyingchi, Pêlung, 1900 m, 24–25.VI.2015, leg. Li Xinxin; 1♂1♀, same locality, 2115 m, 1.IX.2005, leg. Wang Xuejian; 1♀, Nyingchi, Pêlung, Mamba, 2115 m, 1–2.IX.2005, leg. Wang Xuejian; 1♂1♀, Cona, Lexiang, Senmuzha, 2741 m, 2–3.VI.2016, leg. Li Xinxin; 1♀, Cona, Lexiang, Lewangdaqiao, 2423 m, 7.VI.2016, leg. Li Xinxin; 1♂, Yadong, Yadonglinchang, 2690 m, 24.VI.2016, leg. Li Xinxin; 1♂, Gyirong, Resuo, 18.VIII.1984, leg. Pu Qiongqiong; 1♀, Gyirong, Tuowu, 3300 m, 4.VIII.1975, leg. Huang Fusheng; 1♀, Zham, Kouan, 26.IX.1984, leg. Li Aihua; 1♀, Zham, 20.IX.1984, leg. Guo Sengbao, 1♀, Zham, Daqu, 3300 m, 2.VII.1957, leg. Wang Ziqing; 1♀, Zham, Nyalam, 2200 m, 9.V.1966, leg. Wang Shuyong. **Myanmar** (ZFMK): 1♀, Upper Burma Htawgaw, 6000ft, Coll. A.E. Swann.

#### Distribution.

China (Shaanxi, Sichuan, Yunnan, Tibet), India, Myanmar.

#### Remarks.

Chu and Wang (1987) described *Auzatellapentesticha* based on four specimens from Tibet and Hubei Province, and designated the female from Quxam, Tibet as the holotype, the male from Zham, Tibet as the allotype, and another two females from Hubei as paratypes. Unfortunately, the genitalia slide of the female holotype could not be found. In the original description, the figure of the female genitalia is from one of the paratypes from Hubei Province. The only male from Tibet has genitalia identical to those of *D.sericea*, and the two females from Hubei belong to *D.quinaria*. Although the holotype and allotype specimens were collected from two very close localities (Quxam and Zham are less than 30 km apart), and in a very similar season (7 July, 26 June), we hesitate to synonymize *Auzatellapentesticha* Chu & Wang with *Ditrigonasericea* (Leech) without having seen the genitalia of the holotype; however, we redetermine the male allotype as *D.sericea*, and the two paratypes as *Ditrigonaquinariaerminea* Wilkinson.

### ﻿11. Ditrigona pentesticha

Taxon classificationAnimaliaLepidopteraDrepanidae

(Chu & Wang, 1987) comb. nov.

096D591D-77FD-5EFA-8784-DAB8EBC2DEF1

Fig. 10

Auzatella pentesticha Chu & Wang, 1987: 108. Holotype ♀, China: Tibet: Quxam (IZCAS).

#### Material examined.

**China: Tibet** (IZCAS): 1♀, holotype, Quxam, 3300 m, 7.VII.1975, leg. Wang Ziqing.

#### Distribution.

China (Tibet).

#### Remarks.

As stated under the above species, this species now only includes the female holotype. The validity of the species needs further study, for example, by obtaining a DNA barcode from the holotype.

### ﻿12. Ditrigona quinaria

Taxon classificationAnimaliaLepidopteraDrepanidae

(Moore, 1867)

32F1848B-2B2D-59EC-8438-6897C871D8EA

Drepanodes quinaria Moore, 1867: 618. Neotype ♂, India: Darjiling (NHMUK).
Ditrigona quinaria : Wilkinson, 1968: 438.

#### Note.

At present, *D.quinaria* comprises five subspecies; four are recorded from China, the exception being *D.quinarianivea* (Hampson), which is distributed in India.

### ﻿ Ditrigona quinaria quinaria

Taxon classificationAnimaliaLepidopteraDrepanidae

(Moore, 1867)

BB6636DB-4FFE-557D-9836-02B3F826FE6E

#### Material examined.

No.

#### Distribution.

China (Tibet), India.

### ﻿ Ditrigona quinaria erminea

Taxon classificationAnimaliaLepidopteraDrepanidae

Wilkinson, 1968

51A445BD-125D-51A0-91EA-E9A03E632A9B

Figs 11 , 57 , 91 , 124 , 158

Ditrigona quinaria erminea Wilkinson, 1968: 442. Holotype ♂, China: Shaanxi, Tapaishan-im-Tsinling (ZFMK).

#### Material examined.

**China: Shaanxi** (ZFMK): 1♂, holotype, Tapaishan im Tsinling, Sued-Shensi, ca. 3000 m, 26.VI.1936, H. Höne, slide no. 1493, moth photograph examined; 1♂, Tapaishan im Tsinling, Sued-Shensi, China, ca. 3000 m, 11.VIII.1936, H. Höne, dissected in this work; 1♀, same locality and collector, 17.VI.1936, dissected in this work. **Hubei** (IZCAS): 2♀, paratypes of *Auzatellapentesticha* Chu & Wang, 1987, Shennongjia, Jiuhulinchang, 1840 m, 16.VIII.1981, leg. Han Yinheng.

#### Distribution.

China (Shaanxi, Hubei).

### ﻿ Ditrigona quinaria spodia

Taxon classificationAnimaliaLepidopteraDrepanidae

Wilkinson, 1968

6D51511D-A121-5DF4-BB5E-5B4A54CE859E

Figs 12 , 58 , 92 , 125

Ditrigona quinaria spodia Wilkinson, 1968: 442. Holotype ♂, China: Yunnan, A-tun-tse (ZFMK).

#### Material examined.

**China: Yunnan**: 1♂ (ZFMK), holotype, A-tun-tse (N Yünnan), Aus Höhe ca. 4000 m, 25.VII.1937, H. Höne, Drepanidae genitalia slide No. 1487, moth photograph examined; 1♀ (ZFMK), Paratype, A-tun-tse (N. Yünnan), Aus Höhe, ca. 4000 m, 15.VI.1937, H. Höne; 1♂ (ZFMK), paratype, Li-kiang, ca. 3000 m, Prov. Nord-Yuennan, 15.IV.1934, H. Höne; 1♂1♀ (IZCAS), Tengchong, Heinitang, 1930 m, 28–30.V.1992, leg. Xue Dayong; 1♂ (IZCAS), Xianggelila, Gezan, 3141 m, 20–21.VII.2014, leg. Li Xinxin; 1♂ (IZCAS), Weixi, Tacheng, 2800 m, 13–14.VII.2014, leg. Li Xinxin.

#### Distribution.

China (Yunnan).

### ﻿ Ditrigona quinaria leucophaea

Taxon classificationAnimaliaLepidopteraDrepanidae

Wilkinson, 1968

D212121A-7958-533D-A640-6A697BF7B111

Fig. 13

Ditrigona quinaria leucophaea Wilkinson, 1968: 443. Holotype ♂, China: Tibet [Sichuan], Batang (ZFMK).

#### Material examined.

**China: Sichuan**: 1♂ (ZFMK), holotype, Batang (Tibet), Im Tal dea Yantze, ca. 2800 m, 16.V.1936, H. Höne, Drepanidae genitalia slide No. 1486, moth photograph examined.

#### Distribution.

China (Sichuan).

### ﻿13. Ditrigona obliquilinea

Taxon classificationAnimaliaLepidopteraDrepanidae

(Hampson, 1893)

2DD59755-8E7A-5902-9BB9-9D153E77E434

Leucodrepana obliquilinea Hampson, 1893: 333. Lectotype ♂, India: Assam, Naga Hills (NHMUK).
Ditrigona obliquilinea : Wilkinson, 1968: 444.

#### Note.

*Ditrigonaobliquilinea* includes two subspecies, and the nominate subspecies is distributed in India and Myanmar.

**Figures 28–47.**
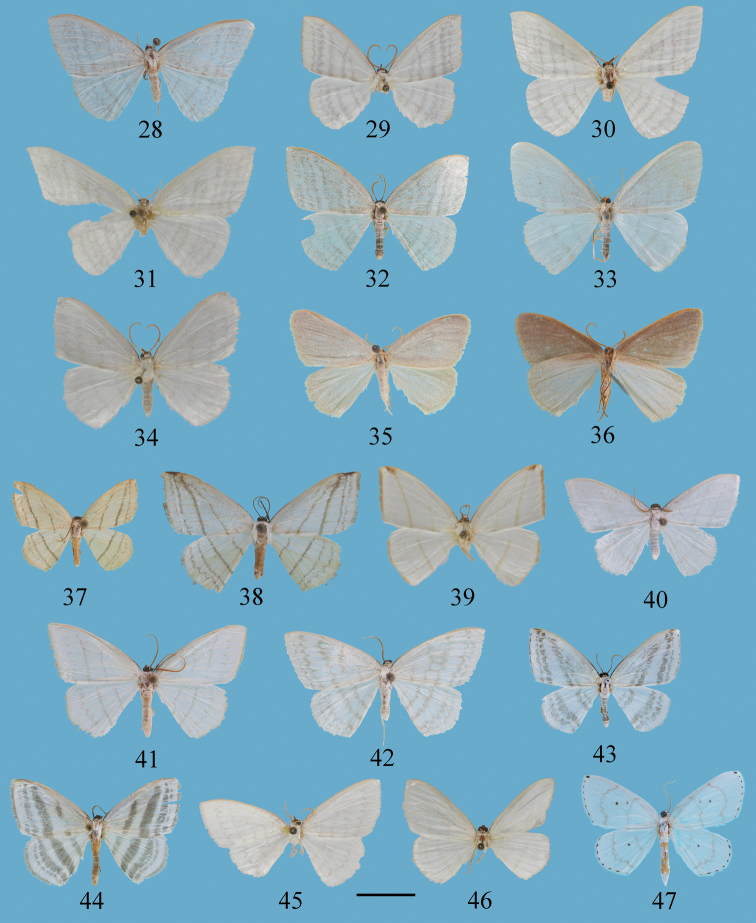
Adults of *Ditrigona***28***D.lineatalineata*, male **29***D.lineatatephroides*, holotype, male, ZFMK**30***D.legnichrysa*, paratype, male, ZFMK**31***D.policharia*, lectotype, female, ZFMK**32***D.artema*, male **33***D.candida*, paratype, male, ZFMK**34***D.chionea*, paratype, male, ZFMK**35, 36***D.fusca* sp. nov., holotype, male **35** upperside **36** underside **37–38***D.conflexariamicronioides***37** male **38** female **39***D.conflexariacerodeta*, holotype, male, ZFMK**40***D.margarita*, male **41***D.berres*, male **42***D.chama*, male **43***D.platytes*, male **44***D.clavata*, male **45***D.marmorea*, paratype, female ZFMK**46***D.aphya*, male **47***D.cirruncata*, male. Scale bar: 1 cm.

### ﻿ Ditrigona obliquilinea thibetaria

Taxon classificationAnimaliaLepidopteraDrepanidae

(Poujade, 1895)

70234F73-EF91-59F8-971B-25F90907ABDB

Figs 14 , 59 , 93 , 126 , 159

Micronia thibetaria Poujade, 1895: 311. Lectotype ♂, China: Thibet [Sichuan], Moupin (MNHN).
Leucodrepana thibetaria : Leech, 1898: 311.
Corycia pnocaria Oberthür, 1923: 238
Ditrigona obliquilinea thibetaria : Wilkinson, 1968: 445.

#### Material examined.

**China: Hunan** (IZCAS): 1♂4♀, Sangzhi, Badagong Shan, Xiaozhuangping, 1420 m, 14.VI.2015, leg. Yao Jian, Zhao Kaidong. **Sichuan**: 2♂ (IZCAS), Jiguan Shan, Shaoyaogou, 1556 m, 11–16.VII.2016, leg. Cui Le; 1♂ (ZFMK), Tien-Tsuen, Yuin-Kin, 1899, Chasseurs indigènes, moth photograph examined. **Tibet** (IZCAS): 1♀, Gyirong, 26.V.1984, leg. Daci; 1♂, Cona, Mama, 2930 m, 18–20.VI.2015, leg. Li Xinxin; 1♂, Cona, Lexiang, Senmuzha, 2741 m, 2–3.VI.2016, leg. Li Xinxin.

#### Distribution.

China (Shaanxi, Zhejiang, Hubei, Hunan, Sichuan, Tibet).

### ﻿14. Ditrigona idaeoides

Taxon classificationAnimaliaLepidopteraDrepanidae

(Hampson, 1893)

D2E3D974-F1A5-577A-8D54-F2BBC4A3ACBC

Leucodrepana idaeoides Hampson, 1893: 333. Lectotype ♂, Sikkim: Tonglo (NHMUK).
Ditrigona idaeoides : Wilkinson, 1968: 447.

#### Material examined.

No

#### Distribution.

China (Sichuan), India.

##### *triangularia* species group

Based on Wilkinson (1968) and Jiang and Han (2019), the *triangularia* species group contains ten species, in which *Ditrigonatriangularia* (Moore), *Ditrigonaregularis* Warren, *Ditrigonauniuncusa* Chu & Wang, and *Ditrigonatenuiata* Jiang & Han bear elongate posterior projections of the hind wings (tail process); the other six species, *Ditrigonatitana* Wilkinson, *Ditrigonapomenaria* (Oberthür), *Ditrigonatyphodes* Wilkinson, *Ditrigonapolyobotaria* (Oberthür), *Ditrigonasciara* Wilkinson, and *Ditrigonafasciata* (Hampson) lack the tail process. The first nine species are recorded in China, and three new species (*D.sinespina*, *D.parva*, *D.concava*) with the tail process are described in this work.

The species with a tail process on the hind wing have quite distinct wing patterns: the hind wing has the postmedial and submarginal lines approaching each other near the anal angle, and bears a small black patch at the upper angle of the tail. The species lacking a tail process resemble some species of the *mytylata* species group, in that they have transverse lines which often resemble a narrow band. In the male genitalia, the valva is characterized by having a small flap-like extension. The species with a tail process can also be distinguished by the large rounded socii and the stout aedeagus bearing a brush-like cornutus. In the species lacking a tail process, the aedeagus is narrow, straight or bent, and the cornutus is a simple process or absent. The eighth sternite is small, shallowly concave or protruding in species with a tail process, and the eighth tergite almost unmodified. Both eighth tergite and sternite often possess octavals in the species lacking a tail process. In the female genitalia, the ostium bursae is usually large, and the ductus bursae is often indiscernible, but wide and obvious in *D.typhodes*. The corpus bursae bears a small accessory sac in species with a tail process. (modified from Wilkinson, 1968)

16 DAN barcoding sequences were obtained for *D.regularis*, *D.triangularia*, *D.tenuiata*, *D.concava* sp. nov., *D.parva* sp. nov., and *D.sinespina* sp. nov., and the six species are clearly separated from each other in the COI barcode fragment (fig. 178). The genetic distance between these species is 8.92% (min. 7.16%, max. 12.32%).

### ﻿15. Ditrigona triangularia

Taxon classificationAnimaliaLepidopteraDrepanidae

(Moore, 1867)

B8178F6E-2F85-55D6-951A-D7FB13189E66

Figs 15 , 60 , 94 , 127

Urapteryx triangularia Moore, 1867: 612. Lectotype ♂, India: Darjiling (NHMUK).
Ditrigona triangularia : Moore, 1888: 258.

#### Material examined.

**India**: 1♂ (NHMUK), lectotype, Darjiling, Moore Coll. 94-106, moth photograph examined. **China: Yunnan**: 1♂ (IZCAS), Weixi, Tacheng, 2800 m, 13–14.VII.2014, leg. Pan Xiaodan; 1♂ (ZFMK), Li-kiang (China), Provinz Nord-Yuennan, 28.VI.1935, H. Höne, moth photograph examined.

#### Distribution.

China (Fujian, Taiwan, Yunnan, Sichuan), India, Myanmar.

### ﻿16. Ditrigona uniuncusa

Taxon classificationAnimaliaLepidopteraDrepanidae

Chu & Wang, 1988

47401DBD-DC3E-5BAA-B33F-42730B8BB522

Figs 16 , 61 , 95 , 128 , 160

Ditrigona uniuncusa Chu & Wang, 1988: 202. Holotype ♂, China: Fujian, Wuyi Shan (IZCAS).

#### Material examined.

**China: Fujian** (IZCAS): 1♂, holotype, Wuyi Shan, 22.VI.1982, leg. Zhang Baolin; 1♀, same locality, 704 m, 12.VIII.1979, leg. Song Shimei; 1♀, Wuyi Shan, Sangang, 704 m, 23.X.1980, leg. Cai Rongquan; 1♀, same locality, 1.VI.1983, leg. Mai Guoqing; 3♀, same locality, 31.VII.2005, leg. Wang Jiashe; 1♂20♀, same locality, 20–21.X.2005, leg. Han Hongxiang, Lang Songyun, Yang Chao; 2♀, same locality, X.1979, leg. Huang Juyi; 1♀, same locality, 8.X.1979, leg. Xu Zhanfei; 1♀, same locality, 15.VI.1981, leg. Jiang Fan; 1♀, same locality, 21.VI.1981, leg. Wang Jiashe, Jiang Fan. **Sichuan** (IZCAS): 1♀, Luding, Moxi, 19–20.V.2009, leg. Li Jing.

**Figures 48–63.**
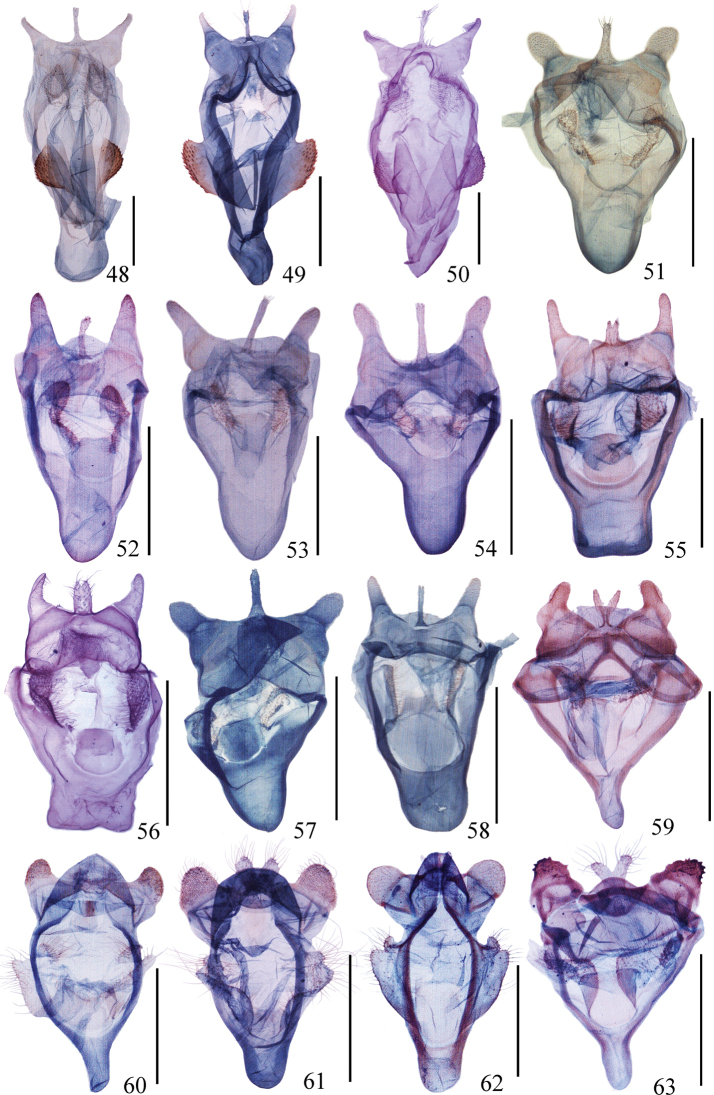
Male genitalia of *Ditrigona***48***D.derocina***49***D.diana***50***D.crystalla*, holotype **51***D.spilota*, ZFMK**52***D.furvicosta***53***D.jardanaria***54***D.media***55***D.sericea***56***D.sericea* (*Auzatellapentesticha* Chu & Wang, allotype) **57***D.quinariaerminea*, ZFMK**58***D.quinariaspodia***59***D.obliquilineathibetaria***60***D.triangularia***61***D.uniuncusa***62***D.tenuiata*, holotype **63***D.regularis*. Scale bars: 1 mm.

#### Distribution.

China (Fujian, Sichuan).

### ﻿17. Ditrigona tenuiata

Taxon classificationAnimaliaLepidopteraDrepanidae

Jiang & Han, 2019

7955CA53-8150-5EE7-B735-2166494381DA

Figs 17 , 62 , 96 , 129 , 161

Ditrigona tenuiata Jiang & Han, 2019: 84. Holotype, ♂, China: Sichuan, Kangding (IZCAS).

#### Material examined.

**China: Sichuan** (IZCAS): 1♂, holotype, Kangding, Xikangyinxiang hotel, 2582 m, 7–10.VIII.2016, leg. Cui Le, M23028; 1♀, paratype, Luding, Hailuogou, 2569 m, 11.IX.2016, leg. Li Xinxin, M25081.

#### Distribution.

China (Sichuan).

### ﻿18. Ditrigona regularis

Taxon classificationAnimaliaLepidopteraDrepanidae

Warren, 1922

F5B4CBD9-BE4F-52E3-A0C2-57A6020D2D40

Figs 18 , 63 , 97 , 130 , 162

Ditrigona regularis Warren, 1922: 463. Lectotype ♂, India: Assam, Khasia (NHMUK).
Ditrigona regularis diflerentiata Bryk, 1943: 9.

#### Material examined.

**China: Guangxi** (IZCAS): 2♀, Napo, Defu, 1350 m, 19.VI.2000, leg. Li Wenzhu. Sichuan: 1♀, Emei Shan, Qingyinge, 800–1000 m, 29.IV.1957, leg. Huang Keren; 2♀, Emei Shan, 0km, 1288 m, 31.VII.2013, leg. Cheng Rui; 1♀, Huili, 19.VII.1974, leg. Han Yinheng; 2♂1♀, Anha, Luoji Shan, 2044 m, 14–16.VII.2018, leg. Cui Le, Jiang Shan. **Yunnan** (IZCAS): 1♀, Pianma, Dianxin hotel, 1970 m, 8–12.V.2011, leg. Yang Xiushuai, Wang Ke; 1♀, Tengchong, Dahaoping, 2020 m, 24–26.V.1992, leg. Xue Dayong; 1♂, same locality, 2020 m, 5–7.VIII.2007, leg. Xue Dayong; 1♀, Tengchong, Shidi, 1730 m, 3–5.VIII.2016, leg. Ban Xiaoshuang; 2♂2♀, Tengchong, Houqiao, 1620 m, 6–8.VIII.2016, leg. Ban Xiaoshuang; 1♀, Lushui, Yaojiaping, 2500 m, 4.VI.1981, leg. Zhang Xuezhong; 1♀, Ruili, Dengga, 980 m, 6–8.VI.1992, leg. Xue Dayong; 2♂3♀, Pingbian, Daweishan, 2090 m, 4–8.VIII.2017, leg. Cui Le; 1♀, Kunming, Shuanglongxiang, 2100 m, 11.VIII.2006, leg. Ma Rong; 1♀, Xinping, Gasa, Yaonan, 1900 m, 10–13.VIII.2017, leg. Cui Le; 1♀, Yunlong, Tianchi Baohuqu, 2570 m, 9–12.VIII.2016, leg. Ban Xiaoshuang. **Tibet** (IZCAS): 1♀, Medôg, 1091 m, 22.VIII.2006, leg. Lang Songyun. **Thailand** (ZFMK): 1♂, Chiangmai Doi Suthep, 1325 m, 21.XI.–4.XII.1989, leg. Schnitzler, moth photograph examined.

#### Distribution.

China (Guangxi, Sichuan, Yunnan, Tibet), India, Myanmar, Thailand.

### ﻿19. Ditrigona sinespina

Taxon classificationAnimaliaLepidopteraDrepanidae

Jiang & Han sp. nov.

5F1DEDAB-BC16-5C0C-A28E-CF9BB7AA6BFC

http://zoobank.org/B95C0171-9D11-419C-B668-371D07B000E3

**Figures 64–74.**
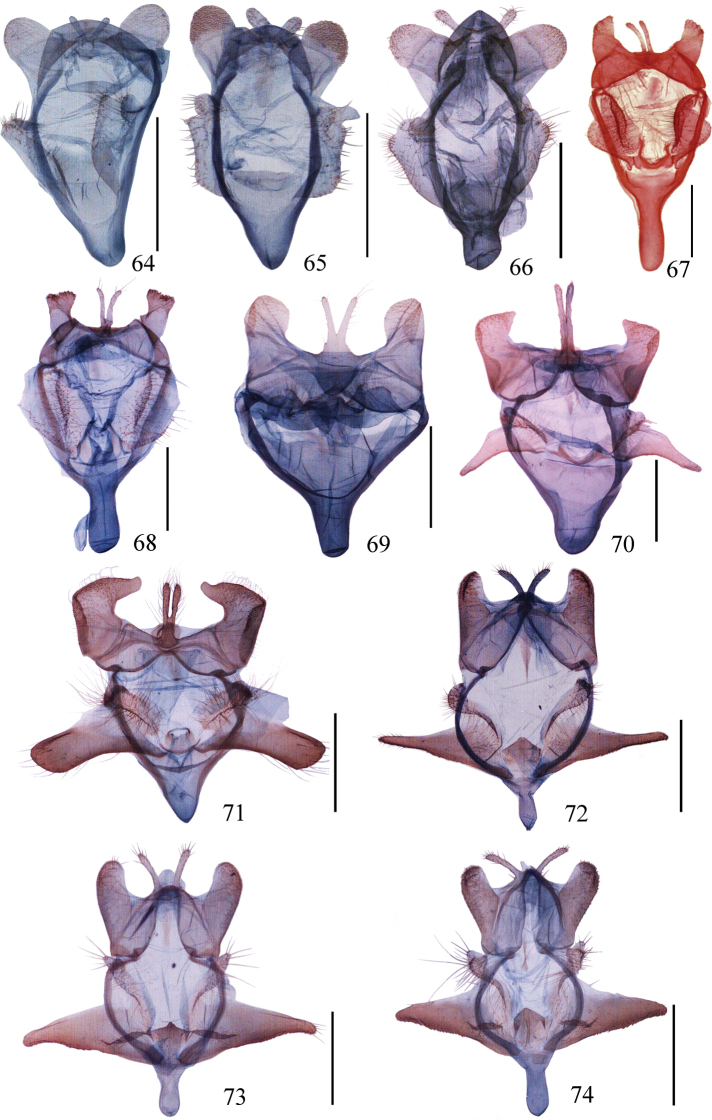
Male genitalia of *Ditrigona***64***D.sinespina* sp. nov., holotype **65***D.parva* sp. nov., holotype **66***D.concava* sp. nov., paratype **67***D.titana*, ZFMK**68***D.pomenaria***69***D.typhodes***70***D.lineatalineata***71***D.artema***72***D.candida*, paratype, ZFMK**73***D.chionea***74***D.fusca* sp. nov., holotype. Scale bars: 1 mm.

Figs 19–20 , 64 , 98 , 131 , 163

#### Description.

***Head*.** Antennae bipectinate, with proximal rami shorter than outer rami, the longest ramus about four times diameter of antennal shaft in male; rami quite short in female, almost equal to diameter of antennal shaft. Frons flattened, width less than diameter of compound eyes; white, upper half with a narrow pale brown transverse band. Labial palpus slender, not extending beyond frons, with outside brown, inner side whitish. Vertex white, pale brown anteriorly.

***Thorax*.** Dorsal and ventral sides of thorax white. Tegula white. Hind tibia with two pairs of spurs in both sexes. Forewing length: ♂♀16 mm. Both fore- and hind wings white, transverse lines grey. Forewing with costa pale brown, distal half deeper. Subbasal and antemedial lines slightly bent inwards at middle and costa, the former narrower; postmedial line broad, almost straight; submarginal line double, the inner one slightly wavy and the outer one deeply wavy. Hind wing with antemedial line straight, merging into the elongate grey area along anal margin; postmedial line broader, almost straight, closing to submarginal line near anal angle, forming large pointed teeth on CuA_2_ and anal fold; submarginal line double, with the inner one nearly straight and only wavy near anal angle, the outer one wavy, the two lines gradually approximating towards anal margin. Anal margin less extended, possessing a quite short tail process, longer in female, with a small black patch. Fringes pale brown. Forewing underside with costa deep brown in basal half.

***Abdomen*.** Dorsal and ventral sides of abdomen white. Eighth tergite large, nearly quadrate, with posterior margin shallowly concave; eighth sternite concave at middle, forming two small lateral blunt processes.

***Male genitalia*.** Uncus bifurcate over its whole length, both halves short and very narrow. Socii large, rounded. Valva small, ventral margin smoothly curved, distal and posterior margins straight, forming a blunt angle; posterior protrusion rounded. Saccus blunt and rounded. Juxta indistinctly shaped. Aedeagus very stout, terminal part narrower; cornutus a large oval spinose patch.

***Female genitalia*.** Papillae analis short; apophyses anteriores moderate, broad basally. Ostium bursae large; ductus bursae indiscernible; corpus bursae round, signum absent.

#### Diagnosis.

On the wing pattern, *D.sinespina* is close to *D.tenuiata*, but it can be differentiated by the larger distance between the two submarginal lines on the forewing, and the smaller tail process. Compared to *D.triangularia* and *D.uniuncusa*, the anal margin of *D.sinespina* is less extended, and the tail process is distinctly shorter than in those two species. Compared to *D.parva* sp. nov. and *D.concava* sp. nov., *D.sinespina* is larger (with forewing length 16 mm), and the tail process on the hind wing is less developed.

In the male genitalia, the slender uncus is similar to that of *D.tenuiata* and *D.concava*, but it is longer than in *D.tenuiata* and shorter than in *D.concava*. The straight distal margin of the valva is also different from these two species. The shape of the aedeagus, which is broad and blunt posteriorly, also can be distinguished from these two species. The eighth tergite of the male is similar to that of *D.concava*, but the eighth sternite is different: in *D.sinespina* it is narrowly and deeply concave, forming two blunt protrusions, while in *D.concava* it is widely and shallowly concave, forming two small lateral processes. The female genitalia of *D.sinespina* are also similar to those of *D.tenuiata*, but can be differentiated by the lack of a signum.

#### Type material.

Holotype, ♂, **China: Yunnan** (IZCAS): Yunlong, Tianchi, 2570 m, 9–12.VIII.2016, leg. Ban Xiaoshuang, slide no. Drep-1054, M33001. Paratypes: **Yunnan** (IZCAS), 1♂, same data as holotype, M33002, posterior part of abdomen missing; 1♂, same data as holotype, M33029; 1♀, Tengchong, Heinitang, 1824 m, 26–27.VI.2014, leg. Pan Xiaodan, slide no. Drep-1060, M33196.

#### Distribution.

China (Yunnan).

#### Etymology.

The species is named from the Latin words *sine* and *spina*, which refers to the lack of a signum in the female genitalia.

#### Molecular data.

The mean intraspecific distance of *D.sinespina* is 1.55% (min. 0%, max. 2.24%, *n* = 4). The nearest related species is *D.tenuiata*, with genetic distance 7.16%.

### ﻿20. Ditrigona parva

Taxon classificationAnimaliaLepidopteraDrepanidae

Jiang & Han sp. nov.

2A49527E-2C9B-5D54-9A86-0AD65B16FC6E

http://zoobank.org/3E199CD5-F712-4F67-B30E-E04C94EA34E9

Figs 21–22 , 65 , 99 , 132 , 164

#### Description.

Head and thorax almost identical to those of *D.sinespina*. Forewing length: ♂11.5 mm, ♀14 mm. Antemedial line almost straight apart from an inward bend at costa. Outer line of the double submarginal lines serrate. Anal margin of hind wing elongate, with relatively large tail process. Fringes brown. Forewing underside with costa brown at basal half.

***Male genitalia*.** Uncus bifurcate over whole length, both sides broad. Socii large, terminally semicircular, scobinate. Valva small, distal margin shallowly concave, posterior protrusion rounded. Saccus blunt and rounded. Juxta with posterior margin almost straight. Aedeagus very stout; cornutus a large oval spinose patch. Eighth tergite quadrate, with a pair of small anterior apodemes; eighth sternite quite small, posterior margin slightly convex.

***Female genitalia*.** Papillae analis short; apophyses anteriores moderate, broad basally. Ostium bursae large; ductus bursae indiscernible; corpus bursae rounded, posteriorly with a large wrinkled sclerotized area and accessory sac, signum short and narrow.

#### Diagnosis.

The wing pattern is very close to that of *D.uniuncusa*. The antemedial line on the hind wing is straight in *D.parva*, but slightly convex in *D.uniuncusa*. The width between the two submarginal lines is larger than in *D.uniuncusa*, especially in the female. In the male genitalia, *D.parva* shares a stout uncus with *D.uniuncusa* and *D.regularis*, but the terminal half of the socii are quite different: scobinate and semicircular in *D.parva*, scobinate and tapering in *D.uniuncusa*, and spinose in *D.regularis*. The female genitalia are also different: the sclerotized area on the corpus bursae is rounded, less sclerotized and smaller than that in *D.uniuncusa*, which has a large oval well sclerotized area; the signum is shorter than in *D.uniuncusa*.

#### Type material.

Holotype, ♂, **China: Yunnan** (IZCAS): Tengchong, Houqiao, 1620 m, 6–8.VIII.2016, leg. Ban Xiaoshuang, slide no. Drep-1057, M33049. Paratypes: 1♂, same data as holotype, M33059; 1♀, same data as holotype, slide no. Drep-1059, M33040; 1♂, same locality, 1553 m, 28–29.VI.2014, leg. Pan Xiaodan.

#### Distribution.

China (Yunnan).

#### Etymology.

The species is named referring to the Latin word *parvus*, which refers to the small wings.

#### Molecular data.

The three specimens of *D.parva* have no genetic distance between them. The nearest related species is *D.concava*, with a genetic distance of 9.56%.

### ﻿21. Ditrigona concava

Taxon classificationAnimaliaLepidopteraDrepanidae

Guo & Han sp. nov.

314F8330-82EE-5C14-82CA-0509F7A6B06E

http://zoobank.org/AB4E8887-3422-4197-AA1C-DC8363354992

Figs 23 , 66 , 100 , 133

#### Description.

Characters of head and thorax in male same as in *D.sinespina*. Forewing length 16 mm in male. Fore- and hind wings white, transverse lines grey. Forewing with costa pale brown. Subbasal line slightly bent inwards at middle and costa; antemedial line straight and only bent inwards near costa; postmedial line broad, almost straight; submarginal line double, the inner one slightly wavy and the outer one deeply wavy. Hind wing with antemedial line slightly convex at middle, bent outwards and merging into the elongate grey area along the anal margin; postmedial line broader, almost straight, slanting outwards and closing to meet submarginal line near anal angle, forming large pointed teeth on CuA_2_ and anal fold; submarginal line double, with the inner one nearly straight and only wavy near anal angle, the outer one wavy, the two lines gradually approximating towards anal margin. Anal margin less extended, with a quite short tail process, longer in the female, with a small black patch. Fringes pale brown. Forewing underside with costa deep brown in basal half.

***Male genitalia*.** Uncus bifurcate over its whole length, both arms slender. Socii large, terminally semicircular, rough apically. Valva small, distal and posterior margin shallowly concave, posterior protrusion rounded. Saccus blunt and rounded. Juxta large, slightly sclerotized, indistinctly shaped. Aedeagus stout, terminal part narrow; cornutus an oblong spinose patch. Eighth tergite quadrate, posterior margin almost straight; eighth sternite shallowly concave, with two small lateral protrusions.

***Female genitalia*.** Unknown.

#### Diagnosis.

The most distinctive character of *D.concava* lies in the male eighth sternite, which is different from all other congeners by the wide and shallow concavity, bearing two small lateral processes. The male genitalia are similar to those of *D.tenuiata* and *D.sinespina*, and can be differentiated by the following differences: the uncus is longer than in those two species; the distal and posterior margins of the valva are shallowly concave in *D.concava*, but the two margins are straight in *D.sinespina*.

#### Type material.

Holotype, ♂, **China: Yunnan** (IZCAS): Ailao Shan, 2600 m, 11.VIII.2011, leg. Kitching Ashton, slide no. Drep-1095, ARB00027811, ex. XTBG. Paratype: 1♂, **Yunnan** (IZCAS): Weixi, Tacheng, 2800 m, 13–14.VII.2014, leg. Li Xinxin, slide no. Drep-1091, M32975.

#### Distribution.

China (Yunnan).

#### Etymology.

The species is named after the Latin word *concavus*, which refers to the shallowly concave 8^th^ male sternite.

#### Molecular data.

An intraspecific distance of *D.sinespina* of 2.07% (*n* = 2) was recorded. The nearest related species is *D.parva*, with a genetic distance of 9.56%.

### ﻿22. Ditrigona titana

Taxon classificationAnimaliaLepidopteraDrepanidae

Wilkinson, 1968

9C77C5BE-01F5-5826-96CC-07412B3E1CF0

Figs 24 , 67 , 101 , 134

Ditrigona titana Wilkinson, 1968: 453. Holotype ♂, China: Yunnan, Likiang (ZFMK).

#### Material examined.

**China: Yunnan**: 1♂ (ZFMK), holotype, Li-kiang, ca. 3000 m, Prov. Nord-Yuennan, 13.IX.1934, H. Höne, moth photograph examined.

#### Distribution.

China (Yunnan).

### ﻿23. Ditrigona sciara

Taxon classificationAnimaliaLepidopteraDrepanidae

Wilkinson, 1968

C1D0FBBF-BAB1-5F34-9AF9-DA67661D5902

Ditrigona sciara Wilkinson, 1968: 458. Holotype ♂, China: Sichuan, Ta-tsien-lou (NHMUK).

#### Material examined.

No.

#### Distribution.

China (Sichuan).

### ﻿24. Ditrigona pomenaria

Taxon classificationAnimaliaLepidopteraDrepanidae

(Oberthür, 1923)

D9B37751-A7AC-56E0-AD24-A4B3E100C6DC

Figs 25 , 68 , 102 , 135

Corycia (Bapta) pomenaria Oberthür, 1923: 238. Lectotype ♂, China: Sichuan, Moupin (ZFMK).
Ditrigona pornenaria : Wilkinson, 1968: 454.

#### Material examined.

**China: Sichuan** (ZFMK): 1♂, holotype, Mou-Pin, 1897, ex. R.P. Déjean, photograph examined; 1♂, Jiguan Shan, Shaoyaogou, 1556 m, 11–16.VII.2016, leg. Cui Le.

#### Distribution.

China (Sichuan).

### ﻿25. Ditrigona polyobotaria

Taxon classificationAnimaliaLepidopteraDrepanidae

(Oberthür, 1923)

9791C487-5320-550B-9AD6-0A0D04C5C3FF

Fig. 26

Corycia polyobotaria Oberthür, 1923: 237. Lectotype ♀, China: Sichuan, Siao-lou (ZFMK).
Ditrigona polyobotaria : Wilkinson, 1968: 458.

#### Material examined.

**China: Sichuan** (ZFMK): 1♀, holotype, Siao-Lou, 1900, Chasseurs indigènes, photograph examined.

#### Distribution.

China (Sichuan).

### ﻿26. Ditrigona typhodes

Taxon classificationAnimaliaLepidopteraDrepanidae

Wilkinson, 1968

F57910E5-D30D-511C-9CF2-CDDEF0304598

Figs 27 , 69 , 103 , 136 , 165

Ditrigona typhodes Wilkinson, 1968: 456. Holotype ♂, China: Yunnan, Likiang (ZFMK).

#### Material examined.

**China: Yunnan**: 1♂ (ZFMK), holotype, Li-kiang, ca. 3000 m, Prov. Nord-Yuennan, 19.VIII.1934, H. Höne, moth photograph examined; 5♂1♀ (IZCAS), Ailao Shan, 2400–2600 m, 6–13.VIII.2011, leg. Ashton, ex. XTBG; 4♂4♀ (IZCAS), Lijiang, 3200–3600 m, 9–14.VIII.2012, leg. Ashton; 2♀ (IZCAS), Lijiang, Yulongshan, 22.VII.–2.VIII.1962, leg. Song Shimei; 1♂ (IZCAS), Lijiang Alpine Botanic Garden, 3272 m, 15–16.VIII.2013, leg. Li Xinxin; 1♀ (IZCAS), Weixi, Tacheng, 2800 m, 13–14.VII.2014, leg. Pan Xiaodan; 1♀ (IZCAS), Lushui, Yaojiaping, 2500 m, 4.VI.1981, leg, Liao Subai; 2♀, Yongsheng, Liude, 2250 m, 10.VII.1984, leg. Liu Dajun. **Sichuan** (IZCAS): 1♂2♀, Yajiang, Bingzhan, 3340 m, 30–31.VII.2014, leg. Li Xinxin.

#### Distribution.

China (Sichuan, Yunnan), Myanmar.

##### *mytylata* species group

In Wilkinson (1968) and Li et al. (2015), 15 species in the *mytylata* species group were recorded in China: *Ditrigonalineata* (Leech), *Ditrigonalegnichrysa* Wilkinson, *Ditrigonapolicharia* (Oberthür), *Ditrigonaartema* Wilkinson, *Ditrigonamarmorea* Wilkinson, *Ditrigonacandida* Wilkinson, *Ditrigonaconflexaria* (Walker), *Ditrigonamargarita* Wilkinson, *Ditrigonaaphya* Wilkinson, *Ditrigonaberres* Wilkinson, *Ditrigonachama* Wilkinson, *Ditrigonachionea* Wilkinson, *Ditrigonaplatytes* Wilkinson, *Ditrigonacirruncata* Wilkinson, and *Ditrigonaclavata* Li & Wang. A new species *D.fusca* is described in this work.

**Figures 75–89.**
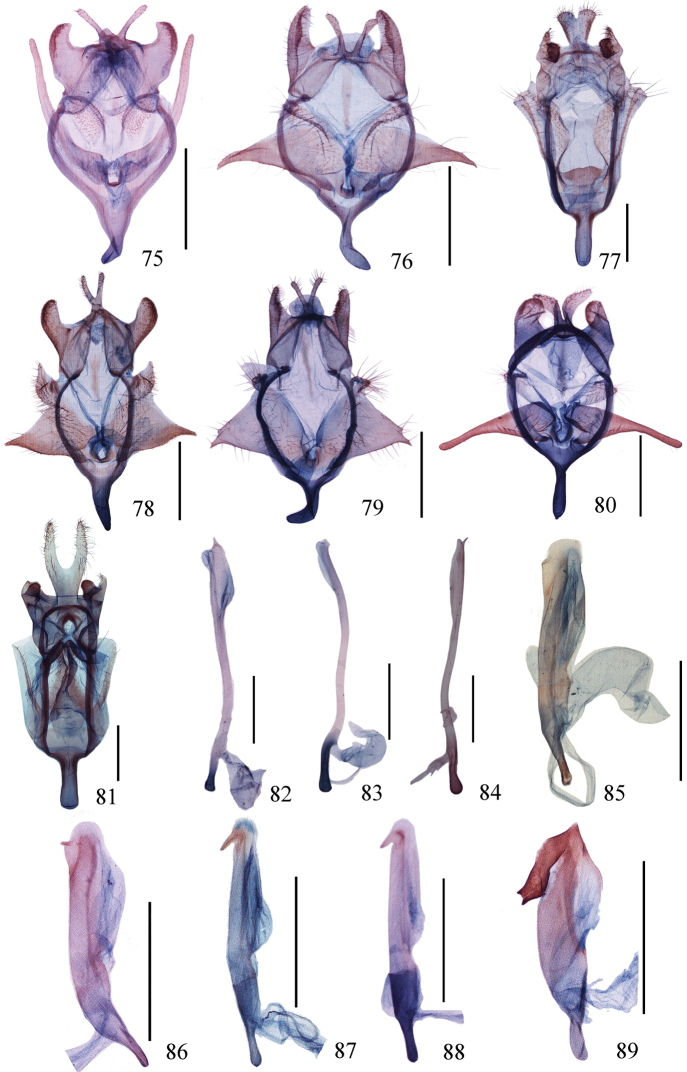
**(75–81)** Male genitalia of *Ditrigona***75***D.conflexariamicronioides***76***D.margarita***77***D.berres***78***D.chama***79***D.platytes***80***D.clavata***81***D.cirruncata*. (**82–89**) Aedeagus of *Ditrigona***82***D.derocina***83***D.diana***84***D.crystalla*, holotype **85***D.spilota*, ZFMK**86***D.furvicosta***87***D.jardanaria***88***D.media***89***D.sericea*. Scale bars: 1 mm.

This species group is characterized by usually having lamellate antennae, and the valva of the male genitalia usually possessing a long posterior extension. Other characters are summarized as follows: the forewing is sometimes weakly falcate; the streaks vary considerably, with transverse lines linear, band-like or absent; the uncus is bifurcate, and the socii are very large; the aedeagus is usually arcuate; both eighth sternite and tergite are modified, the former with short octavals, and the latter concave with small or large protrusions; in the female genitalia, the ostial pocket is characteristic, and the corpus bursae lacks an accessory sac. (modified from Wilkinson, 1968)

### ﻿27. Ditrigona lineata

Taxon classificationAnimaliaLepidopteraDrepanidae

(Leech, 1898)

696F90F2-8D90-5DB3-8DF8-D97D718D7BC4

Leucodrepana lineata Leech, 1898: 364. Holotype ♂, China: Sichuan, Omei-Shan (NHMUK).
Ditrigona lineata : Wilkinson, 1968: 462. Currently, *D.lineata* includes two subspecies, and both are distributed in China.

### ﻿ Ditrigona lineata lineata

Taxon classificationAnimaliaLepidopteraDrepanidae

(Leech, 1898)

C80F7D0B-0E78-5D1C-BE8B-7B7BBE850997

Figs 28 , 70 , 104 , 137 , 166

#### Material examined.

**China: Sichuan** (IZCAS): 1♂, Emei Shan, 15.VIII.1977; 1♂1♀, Emei Shan, Leidongping, 2444 m, 7–8.VIII.2014, leg. Pan Xiaodan; 2♀, Pingwu, Wanglang, Changbaigou, 2480 m, 24.VII.2016, leg. Cui Le. **Yunnan**: 2♂ (IZCAS), Lijiang, Yulong Shan, 30.VII., 4.VIII.1962, leg. Song Shimei; 1♂ (ZFMK) , Li-kiang, China, 6.VIII.1935, Coll. H. Höne.

#### Distribution.

China (Sichuan, Yunnan).

### ﻿ Ditrigona lineata tephroides

Taxon classificationAnimaliaLepidopteraDrepanidae

Wilkinson, 1968

2AC28886-A664-5AD0-9CE7-89E061C3FAEC

Fig. 29

Ditrigona lineata tephroides Wilkinson, 1968: 464. Holotype ♂, China: Shaanxi, Tapaishan-im-Tsinling (ZFMK).

#### Material examined.

**China: Shaanxi** (ZFMK): 1♂, holotype, Tapaishan im Tsinling, Sued-Shensi, ca. 3000 m, 12.VIII.1936, H. Höne, moth and genitalia photos examined.

#### Distribution.

China (Shaanxi, Tibet).

### ﻿28. Ditrigona legnichrysa

Taxon classificationAnimaliaLepidopteraDrepanidae

Wilkinson, 1968

F1AA50E6-52F5-5FA6-A300-65CEE48D17A3

Fig. 30

Ditrigona legnichrysa Wilkinson, 1968: 466. Holotype ♂, China: Tibet [Sichuan] (NHMUK).

#### Material examined.

**China: Sichuan** (ZFMK): 1♂, paratype, Tien-Tsuen, 1897, ex. R.P. Déjean, moth photograph examined.

#### Distribution.

China (Zhejiang, Sichuan, Yunnan, Tibet).

### ﻿29. Ditrigona policharia

Taxon classificationAnimaliaLepidopteraDrepanidae

(Oberthür, 1923)

C9944BFE-38EB-5D7B-A9EC-C17C97367B3C

Fig. 31

Corycia (Bapta) policharia Oberthür, 1923: 237. Lectotype ♀, China: Sichuan, Tsien-Tsuen (ZFMK).
Ditrigona policharia : Wilkinson, 1968: 468.

#### Material examined.

**China: Sichuan** (ZFMK): 1♀, lectotype, Tien-Tsuen, 1897, ex. R. P. Déjean, moth photograph examined.

#### Distribution.

China (Sichuan).

#### Remarks.

This species was described based on a single female specimen only. Wilkinson (1968) stated that the female genitalia are similar to those of *Ditrigonalegnichrysa* Wilkinson, and he also had difficulty in distinguishing it from *D.legnichrysa*, though he listed several tiny differences. He suggested that further material was needed to clarify the relationship between the two species.

### ﻿30. Ditrigona artema

Taxon classificationAnimaliaLepidopteraDrepanidae

Wilkinson, 1968

97C3C3E5-1AC6-569C-AD77-B05F4E5E6882

Figs 32 , 71 , 105 , 138 , 167

Ditrigona artema Wilkinson, 1968: 469. Holotype ♂, China: Sichuan, Siao-lou (NHMUK).

#### Material examined.

**China: Sichuan**: 1♂ (ZFMK), paratype, Ta-tsien-Lou, Chasseurs du P. Déjean, 1904, Ex. Oberthür Coll., Brit. Mus. 1927-3, moth photograph examined; 1♀ (ZFMK), paratype, Wahuipass, 4000 m, Süd. Tatsienlu, VII.–VIII.1930, leg. Friedrich, Coll. Dr. Wehril, moth photograph examined; 1♀ (IZCAS), Emei Shan, 15.VIII.1977; 2♂ (IZCAS), Luding, Hailuogou Erhaoyingdi, 2569 m, 11.IX.2016, leg. Li Xinxin; 1♂6♀ (IZCAS), Luding, Moxi, Hailuogou, 2596 m, 12.IX.2016, leg. Li Xinxin. **Tibet** (IZCAS): 1♂, Yadong, 2760 m, 23–25.VIII.2014, leg. Cheng Rui, Cui Le.

#### Distribution.

China (Sichuan, Tibet).

### ﻿31. Ditrigona candida

Taxon classificationAnimaliaLepidopteraDrepanidae

Wilkinson, 1968

1DB4E8FC-D0D4-56F8-A273-4EA50DB5D948

Figs 33 , 72 , 106 , 139 , 168

Ditrigona candida Wilkinson, 1968: 472. Holotype ♂, China: Yunnan, Likiang (ZFMK).

#### Material examined.

**China: Yunnan** (ZFMK): 1♂, holotype, Li-kiang (China), Provinz Nord-Yuennan, 5.VIII.1935, H. Höne, moth photograph examined; 1♂, paratype, Li-kiang, ca. 2000 m, Prov. Nord-Yuennan, 15.VII.1934, H. Höne, dissected in this work; 1♀, paratype, Li-kiang (China), Provinz Nord-Yuennan, 2.VIII.1935, H. Höne, dissected in this work.

#### Distribution.

China (Yunnan).

### ﻿32. Ditrigona chionea

Taxon classificationAnimaliaLepidopteraDrepanidae

Wilkinson, 1968

9145A8F9-7CDE-530B-821A-36FCA3E08BC0

Figs 34 , 73 , 107 , 140 , 169

Ditrigona chionea Wilkinson, 1968: 490. Holotype ♂, China: ‘Chasseurs Thibetains’ (NHMUK).

#### Material examined.

**China: Yunnan**: 1♂1♀ (ZFMK), paratypes, Li-kiang. ca. 3000 m, Prov. Nord-Yuennan, 24.VII.1934, 14.VI.1934, H. Höne, moth photograph examined; 1♂6♀ (IZCAS), Lijiang Alpine Botanic Garden, 3260–3452 m, 15–20.VI.2009, leg. Xue Dayong, Yang Chao, Han Hongxiang, Qi Feng; 1♀ (IZCAS), Lijiang, Wenhai, 3097 m, 19.VI.2009, leg. Xue Dayong; 1♀ (IZCAS), Lijiang, Ganheba, 3296 m, 23.VI.2009, leg. Qi Feng. **Shaanxi** (IZCAS): 1♀, Zhouzhi, Diaoyutai, 1480 m, 29.VI.2008, leg. Bai Ming.

#### Distribution.

China (Shaanxi, Hubei, Sichuan, Yunnan).

### ﻿33. Ditrigona fusca

Taxon classificationAnimaliaLepidopteraDrepanidae

Guo & Han sp. nov.

D02DAE80-1017-5757-911A-A5AC77C350C5

http://zoobank.org/ED43239E-A79D-4818-B3D3-D4F58984B228

Figs 35–36 , 74 , 108 , 141 , 170

#### Description.

***Head*.** Antennae simple in both male and female. Frons yellow, width less than diameter of compound eyes. Labial palpus with outside deep brown, inner side yellowish. Vertex pale yellow.

***Thorax*.** Tegula pale brown. Dorsal and ventral sides of thorax pale brown. Hind tibia with two pairs of spurs in both sexes. Forewing length: ♂16 mm, ♀15–17 mm. Both wings pale brown, evenly decorated with brown scales, less in basal half of hind wing. Transverse lines absent. Fringes yellowish brown. Underside with forewing deep brown, distal part paler, costa yellowish brown; hind wing with costa yellowish brown, other parts identical to upperside. Fringes yellowish brown.

***Abdomen*.** Dorsal and ventral sides of abdomen pale brown. The eighth tergite with posterior margin concave, with two blunt lateral protrusions; the eighth sternite with posterior margin slightly convex, with two small hooked lateral processes.

***Male genitalia*.** Uncus bifurcate over whole length, the arms narrow and slender. Socii large, tongue-like, of even width, with tips blunt and scobinate. Valva nearly triangular, with tip blunt, ventral margin decorated with tiny spines; basal posterior process bent, tip expanded, with a small accompanying bursa. Juxta rounded, with posterior margin protruding, mound-like. Saccus narrow. Aedeagus slender, almost even in width, tip blunt.

***Female genitalia*.** Ostial pocket band-like. Lamella antevaginalis paired leaf-like. Ductus bursae indiscernible. Corpus bursae rounded; signum a narrow longitudinal sclerotized strip; accessory sac absent.

#### Diagnosis.

The wing pattern is distinctive in lacking transverse lines on both fore- and hind wings. The male genitalia are very close to those of *D.candida*, *D.chionea* and *D.margarita* in the *mytylata* species group, in that they share the slender bifid uncus and tongue-like socii. *D.fusca* and *D.chionea* can be differentiated from those two species by the broader valva, on the base of which a sclerotized ridge is present. The difference between *D.fusca* and *D.chionea* in the male genitalia lies in the shape of the juxta, which is widely protruding posteriorly in *D.fusca*, but only with a tiny process at middle in *D.chionea*. The aedeagus is also different, straight and almost even in width in *D.fusca*, but tapering and twisted in *D.chionea*. The female genitalia of *D.fusca* and *D.chionea* are almost identical.

#### Type material.

Holotype, ♂ (IZCAS), **China: Yunnan**: Lijiang, Yulong Shan, 23.VIII.1962, leg. Song Shimei, slide no. Drep-1092. Paratypes (IZCAS): **Yunnan**: same locality and collector as holotype, 1♂, 3.VIII.1962, slide no. Drep-954; 1♀, 30.VII.1962; 2♀, 2.VIII.1962; 1♀, 3.VIII.1962, 2900 m; 3♀, 4.VIII.1962, slide no. Drep-953; 1♀, 23.VIII.1962; 1♀, 25.VIII.1962; 4♀, 30.VIII.1962; 1♀, Xianggelila, Xiaozhongdian, 3235 m, 15–16.VIII.2016, leg. Ban Xiaoshuang, slide no. Drep-1093; 1♀, Lijiang, Alpine Botanical Garden, 3260–3452 m, 16–18.VI.2009, leg. Qi Feng.

#### Distribution.

China (Yunnan).

#### Etymology.

The specific name is from the Latin word *fuscus*, which refers to the pale brown wing colour.

### ﻿34. Ditrigona conflexaria

Taxon classificationAnimaliaLepidopteraDrepanidae

(Walker, 1861)

F4D6F934-AF90-5EC3-A68E-7BBF686CDD0A

Acidalia conflexaria Walker, 1861: 148. Holotype ♂, N. China (NHMUK).
Ditrigona conflexaria : Wilkinson, 1968: 475. Based on Wilkinson (1968), *D.conflexaria* comprises three subspecies, all distributed in China.

### ﻿ Ditrigona conflexaria conflexaria

Taxon classificationAnimaliaLepidopteraDrepanidae

(Walker, 1861)

DB2F90E3-446D-558B-BDAF-165AC12E5BE6

#### Material examined.

No.

#### Distribution.

North China.

### ﻿ Ditrigona conflexaria micronioides

Taxon classificationAnimaliaLepidopteraDrepanidae

(Strand, 1917)

D78785C4-5306-5814-A290-B0C464A2B89F

Figs 37–38 , 75 , 109 , 142 , 171

Auzata (Auzatella) micronioides Strand, 1917: 148. Holotype ♀, China: Formosa (DEI).
Leucodrepana micronioides : Watson, 1959: 232.
Auzatella micronioides : Inoue, 1962: 12.
Ditrigona conflexaria micronioides : Wilkinson, 1968: 475.

#### Material examined.

**China (IZCAS): Shanxi**: 1♂1♀, Yicheng, Dahelinchang, 1212 m, 13–15.VIII.2018, leg. Zhang Xinyi; 1♀, Yuanqu, Huangguman, 1258 m, 21–22.VIII.2018, leg. Zhang Xinyi. **Henan**: 1♂1♀, Baiyun Shan, 1550 m, 13–15.VIII.2008, leg. Song Wenhui; 1♂1♀, Nanyang, Baotianman, 14.VII.2006, 27.VII.2006, leg. Shen Xiaocheng et al.; 13♂1♀, Baotianman, 1407 m, 10–11.VIII.2008, leg. Jiang Nan, Song Wenhui, Xue Dayong. **Shaanxi**: 1♂, Shangnan, Jinsixia, 777 m, 23–25.VII.2013, leg. Jiang Nan; 5♂, same locality, 766 m, 16–19.VII.2017, leg. Cui Le; 3♂, Ningshan, Yueba, 1052 m, 1–3.VIII.2018, leg. Zhang Xinyi; 3♂, Mei Xian, Honghegu, Shenxianling, 1239 m, 21–22.VII.2018, leg. Zhang Xinyi; 2♀, same locality, 874 m, 20.VII.2018, leg. Zhang Xinyi; 6♂, Foping, Longcaoping, 1218 m, 4.VIII.2018, leg. Zhang Xinyi; 4♂, Taibai, Huangbaiyuan, 1279 m, 15–17.VII.2018, leg. Zhang Xinyi. **Zhejiang**: 5♂, Zhoushan, Putuo, Taohuadao, 40 m, 4.VIII.2016, leg. Li Xinxin; 1♂, Taishun, Wuyanling, Shangfengxiang, 1050 m, 30.VII.2005, leg. Lang Songyun. **Hubei**: 8♂4♀, Lichuan, Xingdoushan, Sanxianchang, 1144 m, 17–19.V.2017, leg. Li Henan; 5♂, Xuanen, Changtanhe, Lianghekou, 949 m, 13–14.V.2017, leg. Li Henan; 5♂2♀, Xuanen, Changtanhe, Dawolong, 713–794 m, 15–16.V.2017, leg. Li Henan; 1♀, Ying Shan, Wujiashan, 500 m, 30.VI.2014, leg. Xue Dayong. **Jiangxi**: 1♂1♀, Jinggangshan, Huangyangjie, 1090 m, 4.VIII.2013, leg. Pan Xiaodan. **Hunan**: 3♀, Zhangjiajie, Wulingyuan, Wenfeng, 475 m, 10.VI.2015, leg. Yao Jian, Zhao Kaidong; 1♂1♀, Yanling, Taoyuandong, 631 m, 4–8.VII.2008, leg. Chen Fuqiang. **Fujian**: 1♂, Meihua Shan, Huyuan, 1276 m, 19.VII.2013, leg. Xue Dayong; 1♀, Masu, 25.IX.1981, leg, Jiang Fan. **Guangxi**: 1♀, Maoershan, Jiuniuchang, 1150 m, 7.VII.1985, leg. Fang Chenglai; 1♀, Maoershan, Jiuniutang, 1146 m, 16.VIII.2012, leg. Yang Chao; 1♂, Huanjiang, Yangmeiao, 1189 m, 18–22.VII.2015, leg. Jiang Nan. **Sichuan**: 1♂, Anha, Luojishan, 2044 m, 14–16.VII.2018, leg. Cui Le, Jiang Shan; 1♂, Hongya, Wawushan, Jinhuaqiao, 1147 m, 12–14.VIII.2016, leg. Cui Le; 12♂6♀, Emei Shan, Qingyinge, 800–1000 m, 16.IV., 17.IV., 19.IV., 24.IV., 26.IV., 27.IV., 29.IV., 30.IV., 12.V., 17.V., 18.VII.1957, leg. Huang Keren, Zhu Fuxing, Lu Youcai, Wang Zongyuan; 1♂, Emei Shan, 1100 m, 22.VI.1955, leg. Li Jinhua; 2♂, Wan Xian, Wangerbao, 1200 m, 27.IX.1994, leg. Song Shimei; 1♂ (ZFMK), Ost Tien-mu-shan, Chekiang, 14.VII.1931, H. Höne, moth photograph examined. **Chongqing**: 1♀, Jinyun Shan, 800 m, 13.VI.1994, leg. Li Wenzhu; 2♂4♀, Chongqing, 800 m, 20.VI., 22.VI.1974, leg. Han Yinheng. **Guizhou**: 2♀, Qianping Shan, Fudingshan, 604 m, 1–4.V.2018, leg. Zhao Kaidong.

**Figures 90–106.**
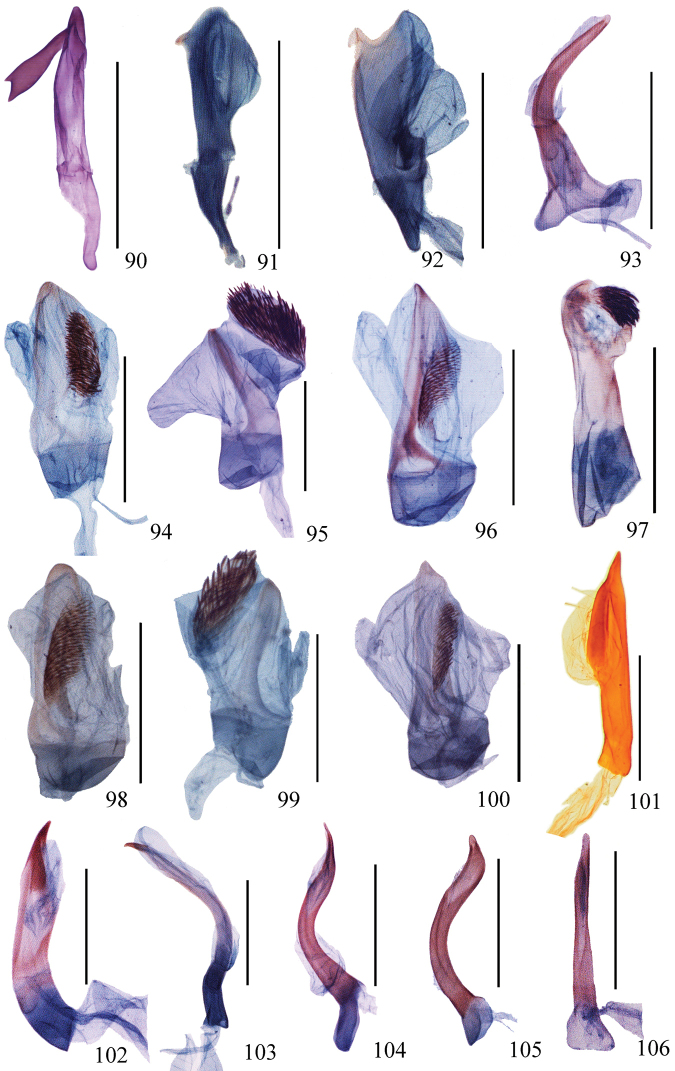
Aedeagus of *Ditrigona***90***D.sericea* (*Auzatellapentesticha* Chu & Wang, allotype) **91***D.quinariaerminea*, ZFMK**92***D.quinariaspodia***93***D.obliquilineathibetaria***94***D.triangularia***95***D.uniuncusa***96***D.tenuiata*, holotype **97***D.regularis***98***D.sinespina* sp. nov., holotype **99***D.parva* sp. nov., holotype **100***D.concava* sp. nov., paratype **101***D.titana*, ZFMK**102***D.pomenaria***103***D.typhodes***104***D.lineatalineata***105***D.artema***106***D.candida*, ZFMK. Scale bars: 1 mm.

#### Distribution.

China (Shanxi, Henan, Shaanxi, Zhejiang, Hubei, Jiangxi, Hunan, Fujian, Taiwan, Guangxi, Sichuan, Chongqing, Guizhou), Japan.

### ﻿ Ditrigona conflexaria cerodeta

Taxon classificationAnimaliaLepidopteraDrepanidae

Wilkinson, 1968

0B7DC158-3718-5B87-A558-665F0090B7AF

Fig. 39

Ditrigona conflexaria cerodeta Wilkinson, 1968: 477. Holotype ♂, China: Likiang (ZFMK).

#### Material examined.

**China: Yunnan**: 1♂ (ZFMK), holotype, Li-kiang. ca. 3000 m, Prov. Nord-Yuennan, 8.VIII.1934, H. Höne, moth photograph examined; 1♀ (ZFMK), paratype, Li-kiang. ca. 3000 m, 4.VIII.1934, H. Höne, moth photograph examined.

**Figures 107–127.**
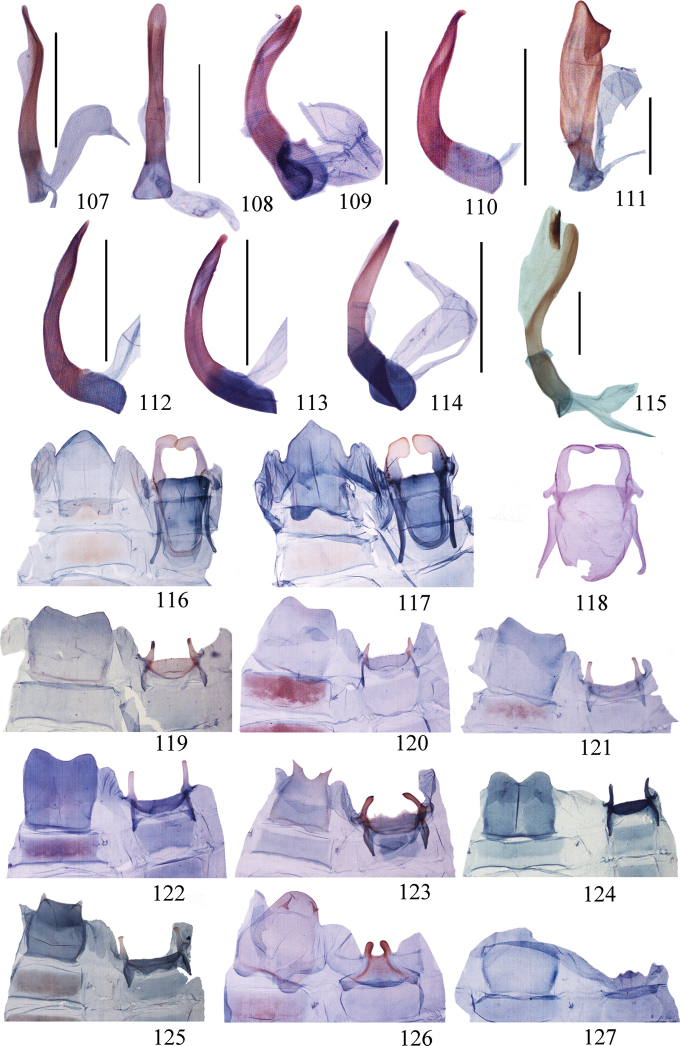
(**107–115**) Aedeagus of *Ditrigona***107***D.chionea***108***D.fusca* sp. nov., holotype **109***D.conflexariamicronioides***110***D.margarita***111***D.berres***112***D.chama***113***D.platytes***114***D.clavata***115***D.cirruncata*. Scale bars: 1 mm. (**116–127)** Eighth segment of *Ditrigona***116***D.derocina***117***D.diana***118***D.crystalla*, holotype **119***D.spilota*, ZFMK**120***D.furvicosta***121***D.jardanaria***122***D.media***123***D.sericea***124***D.quinariaerminea*, ZFMK**125***D.quinariaspodia***126***D.obliquilineathibetaria***127***D.triangularia*

#### Distribution.

China (Yunnan).

**Figures 128–151.**
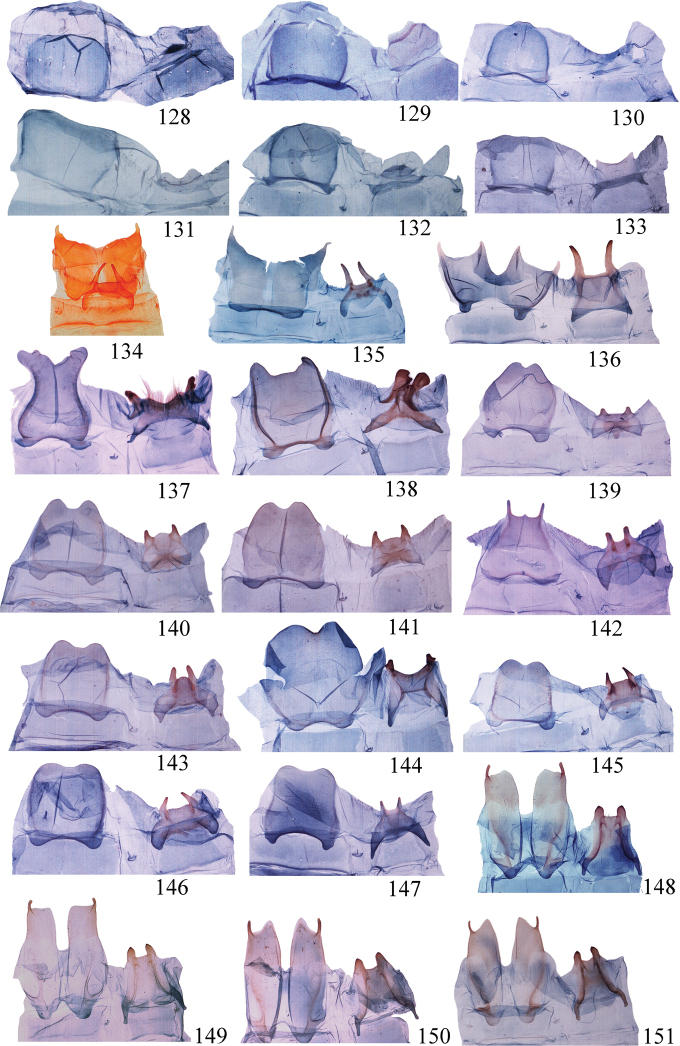
Eighth segment of *Ditrigona***128***D.uniuncusa***129***D.tenuiata*, holotype **130***D.regularis***131***D.sinespina* sp. nov., holotype **132***D.parva* sp. nov., holotype **133***D.concava*, paratype **134***D.titana*, ZFMK**135***D.pomenaria***136***D.typhodes***137***D.lineatalineata***138***D.artema***139***D.candida*, ZFMK**140***D.chionea***141***D.fusca*, sp. nov., holotype **142***D.conflexariamicronioides***143***D.margarita***144***D.berres***145***D.chama***146***D.platytes***147***D.clavata***148–151***D.cirruncata***148** from Shaanxi **149–150** from Emei Shan, Sichuan **151** from Zhejiang.

### ﻿35. Ditrigona margarita

Taxon classificationAnimaliaLepidopteraDrepanidae

Wilkinson, 1968

9EA267DE-928A-548E-A3CD-0F7D0BEDE139

Figs 40 , 76 , 110 , 143 , 172

Ditrigona margarita Wilkinson, 1968: 483. Holotype ♂, China: Shaanxi, Tapaishan-im-Tsinling (ZFMK).

#### Material examined.

**China: Shaanxi**: 1♂ (ZFMK), holotype, Tapaishan-im-Tsinling, Sued-Shensi. ca. 1700 m, 22.VI.1936, H. Höne, moth photograph examined; 1♂ (IZCAS), Ningshan, Huoditang, 1538 m, 11–15.VII.2012, leg. Cheng Rui; 5♂ (IZCAS), Nanzheng, Liping, 1540 m, 27–30.VII.2017, leg. Li Henan. **Henan** (IZCAS): 1♂, Baotianman, 27.VII.2006, leg. Shen Xiaocheng et al. **Ningxia** (IZCAS): 1♂, Jingyuan, Qiuqianjialinchang, 1822 m, 23.VI.2008, leg. Song Wenhui; 1♀, Jingyuan, Erlonghe, 1984 m, 11–12.VII.2008, leg. Song Wenhui. **Gansu** (IZCAS): 1♀, Kang Xian, Qinghelinchang, 1400 m, 8.VII.1999, leg. Zhu Chaodong; 1♀, Zhouqu, Shatanlinchang, 2400 m, 14.VII.1999, leg. Yao Jian; 1♀, Wen Xian, Qiujiaba, 2350 m, 21.VII.1999, leg. Zhu Chaodong; 1♀, same locality and date, leg. Yao Jian. **Sichuan** (IZCAS): 1♂, Mao Xian, 9–11.VII.2015, leg. Li Xinxin; 1♀, Jiguan Shan, Baliping, 1470 m, 15–16.VII.2016, leg. Cui Le; 1♂, Jiguan Shan, Shaoyaogou, 1556 m, 11–16.VII.2016, leg. Cui Le; 2♀, Pingwu, Wanglang, Qikeshu, 2446 m, 21–22.VII.2016, leg. Cui Le.

#### Distribution.

China (Shanxi, Henan, Shaanxi, Ningxia, Gansu, Sichuan).

### ﻿36. Ditrigona berres

Taxon classificationAnimaliaLepidopteraDrepanidae

Wilkinson, 1968

836B9D84-BB1F-515A-AE2A-C0EBC1AE4C23

Figs 41 , 77 , 111 , 144 , 173

Ditrigona berres Wilkinson, 1968: 486. Holotype ♂, China: Shaanxi, Tapaishan-im-Tsinling (ZFMK).

#### Material examined.

**China: Shaanxi**: 1♂ (ZFMK), holotype, Tapaishan-im-Tsinling Sued-Shensi, ca. 3000 m, 23.VI.1936, H. Höne, moth photograph examined; 1♂ (IZCAS), Feng Xian, Jialingjiangyuantou, 1510 m, 21–24.VII.2017, leg. Cui Le. **Hubei** (IZCAS): 2♀, Shennongjia, Dajiuhu, 1800 m, 1.VIII.1981, leg. Han Yinheng; 1♂, Xingshan, Longmenhe, 1300 m, 14.VI.1993, leg. Li Hongxing. **Hunan** (IZCAS): 2♂1♀, Sangzhi, Badagongshan, Xiaozhuangping, 1420 m, 18.VI.2015, leg. Yao Jian, Zhao Kaidong.

#### Distribution.

China (Shaanxi, Hubei, Hunan).

### ﻿37. Ditrigona chama

Taxon classificationAnimaliaLepidopteraDrepanidae

Wilkinson, 1968

9B3E5ABD-EDC6-53A4-BD63-3259BAE83A9D

Figs 42 , 78 , 112 , 145 , 174

Ditrigona chama Wilkinson, 1968: 488. Holotype ♂, China: Sichuan, Siao-lou (NHMUK).

#### Material examined.

**China: Yunnan**: 1♂ (ZFMK), paratype, Li-kiang. ca. 3000 m, Prov. Nord-Yuennan, 28.VII.1934, H. Höne, moth photograph examined; 2♂ (IZCAS), Yongsheng, Liude, 2250 m, 10.VII.1984, leg. Liu Dajun; 1♂ (IZCAS), Tengchong, Qushi, Dabacun, 1873 m, 4.VII.2013, leg. Liu Shuxian. **Shanxi** (IZCAS): 15♂14♀, Pangquangou, Erhezhuang, 1670 m, 4–6.VII.2018, leg. Cui Le, Jiang Shan. **Shaanxi** (IZCAS): 1♂, Mei Xian, Honghegu, Shenxianling, 1239 m, 21–22.VII.2018, leg. Zhang Xinyi; 1♂, Ningshan, Huoditang, 1497 m, 29–31.VII.2018, leg. Zhang Xinyi; 1♀, Zhouzhi, Diaoyutai, 1480 m, 29.VI.2008, leg. Bai Ming; 1♂, Foping, Longcaoping, 1256 m, 3.VII.2008, leg. Liu Wangang, Cui Junzhi; 2♂, Taibai, Huangbaiyuan, 1323 m, 17–18.VI.2012, leg. Li Jing, Liu Shuxian; 1♂1♀, Feng Xian, Jialingjiangyuantou, 1510 m, 21–24.VII.2017, leg. Cui Le; 1♂1♀, Zhashui, Yingpanzhen, Niubeiliang, Laolin, 1046 m, 11–15.VII.2017, leg. Cui Le. **Gansu** (IZCAS): 1♂, Kang Xian, Qinghelinchang, 1400 m, 7.VII.1999, leg. He Tongli, Yao Jian; 1♂, Zhouqu, Shatanlinchang, 2350 m, 5.VII.1998, leg. Yuan Decheng. **Zhejiang**: 2♀, Tianmu Shan, 26.VI.1957, leg. Su Jiyao. **Sichuan** (IZCAS): 1♀, Emei Shan, Jiulaodong, 1800–1900 m, 6.VII.1957, leg. Zhu Fuxing; 1♀, Jiguan Shan, Shaoyaogou, 1556 m, 11–16.VII.2016, leg. Cui Le.

#### Distribution.

China (Shanxi, Shaanxi, Gansu, Zhejiang, Hubei, Sichuan, Yunnan, Tibet).

### ﻿38. Ditrigona platytes

Taxon classificationAnimaliaLepidopteraDrepanidae

Wilkinson, 1968

B9FEC723-9FC6-5996-AF9A-FD26465AF029

Figs 43 , 79 , 113 , 146 , 175

Ditrigona platytes Wilkinson, 1968: 492. Holotype ♂, China: Chekiang, West Tien-mu-Shan (ZFMK).

#### Material examined.

**China: Zhejiang**: 1♂ (ZFMK), holotype, West Tien-mu-shan, Prov. Chekiang, 29.V.1932, H. Höne, moth photograph examined; 1♂ (IZCAS), Tianmu Shan, 26.VI.1957, leg. Su Jiyao; 2♂ (IZCAS), West Tianmu Shan, Qianmutian, 1330 m, 30.VII.2011, leg. Yan Keji, Cheng Rui. **Fujian**: 1♀ (ZFMK), paratype, Kuatun, 2300 m, 26.V.1938, leg. J. Klapperich, moth photograph examined. **Shaanxi** (IZCAS): 2♂, Ningshan, Huoditang, 1538 m, 11–15.VII.2012, leg. Yang Xiushuai, Cheng Rui; 1♂, same locality, 1520 m, 13–17.VIII.2016, leg. Cheng Rui, Jiang Shan; 4♂5♀, same locality, 1497 m, 29–31.VII.2018, leg. Zhang Xinyi; 2♂, Baoji, Jialingjiangyuantou, 1620 m, 8–9.VII.2014, leg. Xue Dayong, Ban Xiaoshuang; 2♂1♀, Nanzheng, Liping, 1540 m, 27–30.VII.2017, leg. Li Henan; 1♂, Zhashui, Yingpanzhen, Niubeiliang, Laolin, 1046 m, 11–15.VII.2017, leg. Cui Le; 8♂7♀, Zhashui, Yingpanzhen, Niubeiliang, 1373 m, 24–26.VII.2018, leg. Zhang Xinyi; 1♂, Foping, Longcaoping, 1218 m, 4.VIII.2018, leg. Zhang Xinyi. **Hubei** (IZCAS): 1♂, Yichang, Dengcunxiang, Dalaoling; 1709 m, 15.VII.2013, leg. Cheng Rui. **Sichuan** (IZCAS): 1♀, Emei Shan, Jiulaodong; 1800–1900 m, 28.VII.1957, leg. Huang Keren; 1♂, Baoxing, Dashuigou, 1591 m, 1–5.VIII.2016, leg. Cui Le; 1♂1♀, Jiguan Shan, Shaoyaogou, 1556 m, 11–16.VII.2016, leg. Cui Le. **Yunnan** (IZCAS): 4♂, Pingbian, Dawei Shan, 2090 m, 4–8.VIII.2017, leg. Cui Le.

**Figures 152–160.**
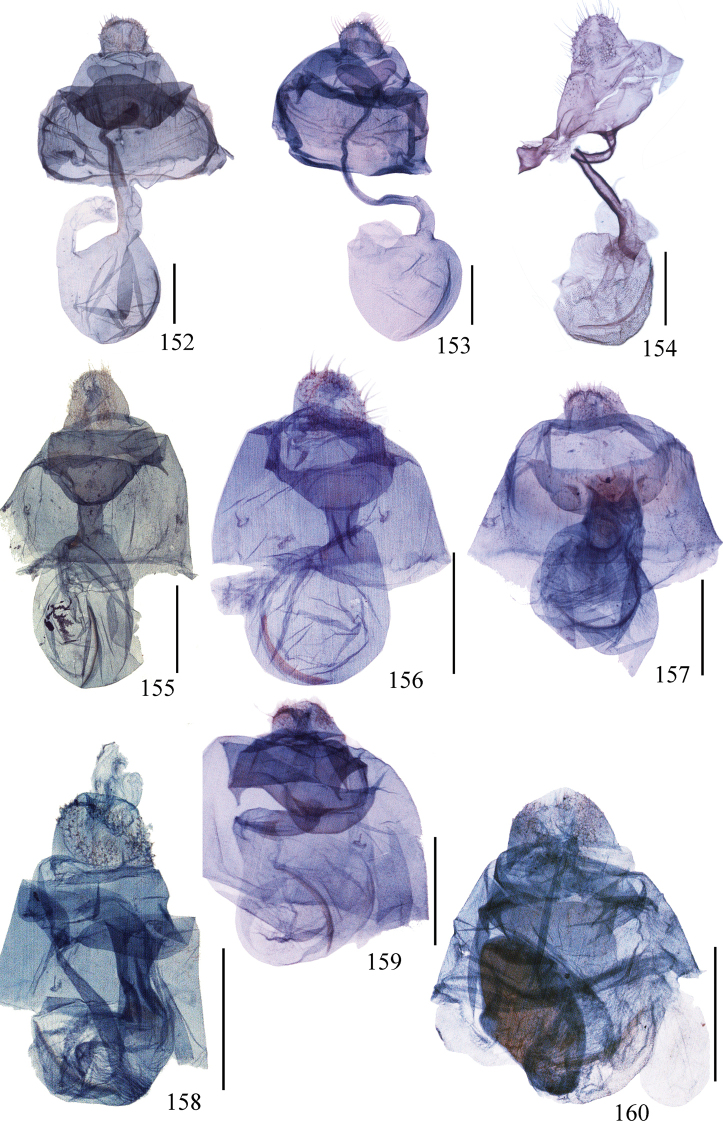
Female genitalia of *Ditrigona***152***D.derocina***153***D.diana***154***D.crystalla*, allotype **155***D.spilota*, ZFMK**156***D.furvicosta***157***D.sericea***158***D.quinariaerminea*, ZFMK**159***D.obliquilineathibetaria***160***D.uniuncusa*. Scale bars: 1 mm.

#### Distribution.

China (Shaanxi, Zhejiang, Hubei, Fujian, Sichuan, Yunnan).

### ﻿39. Ditrigona clavata

Taxon classificationAnimaliaLepidopteraDrepanidae

Li & Wang, 2015

51B31D3A-C2EF-51B4-B324-AB48C4CA6043

Figs 44 , 80 , 114 , 147 , 176

Ditrigona clavata Li & Wang, 2015: 567. Holotype ♂, China: Guangxi, Mao’ershan National Nature Reserve (SCAU).

#### Material examined.

**China: Shaanxi** (IZCAS): 1♂, Liuba, Chengguanzhen, 1007 m, 21–22.VI.2012, leg. Li Jing; 1♂, same locality, 966 m, 23.VI.2012, leg. Liu Shuxian; 1♂, Yang Xian, Huayangzhen, 1099–1108 m, 25–27.VI.2012, leg. Li Jing; 7♂1♀, Ningshan, Yueba, 1052 m, 1–3.VIII.2018, leg. Zhang Xinyi; 2♂, Kang Xian, Qinghelinchang, 1400 m, 8.VII.1999, leg. Zhu Chaodong. **Guangxi** (IZCAS): 2♂1♀, Huanjiang, Yangmeiao, 1189 m, 18–22.VII.2015, leg. Jiang Nan, Li Xinxin.

#### Distribution.

China (Shaanxi, Gansu, Guangdong, Guangxi).

### ﻿40. Ditrigona marmorea

Taxon classificationAnimaliaLepidopteraDrepanidae

Wilkinson, 1968

0BBA253E-2CE2-5372-A871-9A5D0D73F92E

Fig. 45

Ditrigona marmorea Wilkinson, 1968: 471. Holotype ♂, Assam: Mishmi Hills (NHMUK).

#### Material examined.

**China: Yunnan** (ZFMK): 1♂, paratype, Li-kiang (China), Provinz Nord-Yuennan, 25.VI. 1935, H. Höne, moth photograph examined.

#### Distribution.

China (Yunnan), India.

### ﻿41. Ditrigona quinquelineata

Taxon classificationAnimaliaLepidopteraDrepanidae

(Leech, 1898)

6F267BB9-AB6A-5B59-9BDC-B5B99B3563B7

Leucodrepana quinquelineata Leech, 1898: 364. Holotype ♂, Japan (NHMUK).
Auzatella quinquelineata : Inoue, 1962: 13.
Ditrigona quinquelineata : Wilkinson, 1968: 480.

#### Material examined.

No.

#### Distribution.

China (Sichuan), Japan.

### ﻿42. Ditrigona aphya

Taxon classificationAnimaliaLepidopteraDrepanidae

Wilkinson, 1968

751C930A-DD07-5B98-A5BF-C28B3581AE70

Fig. 46

Ditrigona aphya Wilkinson, 1968: 485. Holotype ♂, China: Shaanxi, Tapaishan-im-Tsinling (ZFMK).

#### Material examined.

**China: Shaanxi** (ZFMK) : 1♂, holotype, Tapaishan-im-Tsinling Sued-Shensi, ca. 1700 m, 20.VI.1936, H. Höne, moth photograph examined.

**Figures 161–172.**
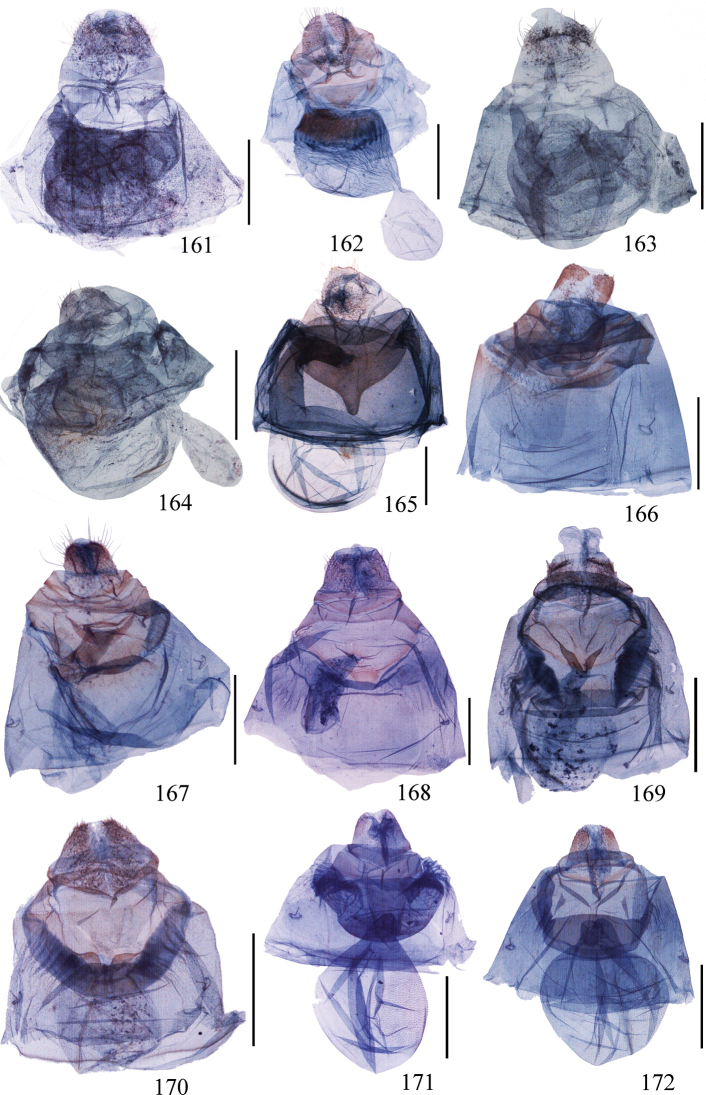
Female genitalia of *Ditrigona***161***D.tenuiata*, paratype **162***D.regularis***163***D.sinespina* sp. nov., paratype **164***D.parva* sp. nov., paratype **165***D.typhodes***166***D.lineatalineata***167***D.artema***168***D.candida*, paratype, ZFMK**169***D.chionea***170***D.fusca* sp. nov., paratype **171***D.conflexariamicronioides***172***D.margarita*. Scale bars: 1 mm.

#### Distribution.

China (Shaanxi).

### ﻿43. Ditrigona cirruncata

Taxon classificationAnimaliaLepidopteraDrepanidae

Wilkinson, 1968

70E6AD49-5BB6-5621-86B7-08B18F318967

Figs 47 , 81 , 115 , 148–151 , 177

Ditrigona cirruncata Wilkinson, 1968: 497. Holotype ♂, China: Sichuan, Siao-lou (ZFMK).

#### Material examined.

**China: Sichuan**: 1♂ (ZFMK), paratype, Kwanhsien Dist. Suchwan, 16.VIII.1925, leg. G.M. Franck, moth photograph examined; 31♂14♀ (IZCAS), Emei Shan, Qingyinge, 800–1000 m, 14.IV., 16.IV., 17.IV., 18.IV., 19.IV., 24.IV., 25.IV., 26.IV., 27.IV., 29.IV., 30.IV., 1.V., 2.V., 5.V., 6.V., 20.VI., 29.VI., 16.VIII., 18.IX., 20.IX.1957, leg. Wang Zongyuan, Zhu Fuxing, Huang Keren, Lu Youcai; 3♂ (IZCAS), Jiulong Shan, Shizipo, 810 m, 29–31.VII.2016, leg. Cui Le; 2♀, Baoxing, Dashuigou, 1591 m, 1–5.VIII.2016, leg. Cui Le; 1♂2♀ (IZCAS), Hongya, Wawu Shan, Jinhuaqiao, 1147 m, 12–14.VIII.2016, leg. Cui Le; 1♂5♀ (MHBU), Emei Shan, 17–19.IX.2010, leg. Niu Yiping. **Shanxi** (IZCAS): 1♂, Qinshui, Manghe, 557 m, 19–20.VIII.2018, leg. ZhangXinyi. **Henan** (IZCAS): 1♂, Baotianman, 1407 m, 10–11.VIII.2008, leg. Jiang Nan. **Shaanxi**: 11♂17♀ (IZCAS), Ningshan, Huoditang, 1520 m, 13–17.VIII.2016, leg. Cheng Rui, Jiang Shan; 3♂9♀ (IZCAS), same locality, 1497 m, 29–31.VII.2018, leg. Zhang Xinyi; 1♂ (IZCAS), Ningshan, Yueba, 1052 m, 1–3.VIII.2018, leg. Zhang Xinyi; 1♂ (IZCAS), Zhashui, Yingpanzhen, Niubeiliang, 1373 m, 24–26.VII.2018, leg. Zhang Xinyi; 1♀ (IZCAS), Foping, Longcaoping, 1218 m, 4.VIII.2018, leg. Zhang Xinyi; 1♀ (MHBU), Ningshan, Huoditang, 1505 m, 14.VIII.2013, leg. Zhu Xichao, Tian Ying; 1♀ (MHBU), Ningshan, Guanghuojie, 1135 m, 10.VIII.2013, leg. Zhu Xichao, Tian Ying. **Gansu** (IZCAS): 3♀, Wen Xian, VI.–IX.2002, leg. Wang Hongjian; 3♂, Bikou, Bifenggou, 720 m, 8–10.VIII.2016, leg. Cheng Rui, Jiang Shan. **Zhejiang** (IZCAS): 3♂, Lin’an, West Tianmushan, 400 m, 26–27.VII.2003, leg. Xue Dayong, Han Hongxiang; 2♀, West Tianmushan, Xianrending, 1506 m, 27.VII.2011, leg. Yan Keji, Cheng Rui; 1♀, West Tianmushan, Qianmutian, 1330 m, 30.VII.2011, leg. Yan Keji, Cheng Rui; 1♂1♀, Yuyao, Simingshan, 809–853 m, 22–22.VII.2015, leg. Cheng Rui. **Anhui**: 1♀ (MHBU), Shitai, Shanshan, 7.VIII.2010, leg. Ba Yibin, Zhang Zhenxing. **Hubei** (IZCAS): 2♀, Shennongjia, Honghua, 860 m, 21.VIII.1981, leg. Han Yinheng; 1♂, Ying Shan, Taohuachong, 590 m, 23–27.VI.2014, leg. Jiang Nan; 4♂, Xuanen, Changtanhe, Lianghekou, 949 m, 13–14.V.2017, leg. Li Henan; 1♀, Xuanen, Changtanhe, Dawolong, 713 m, 15.V.2017, leg. Li Henan; 2♂1♀, same locality and collector, 794 m, 16.V.2017, leg. Li Henan; 1♀, Lichuan, Xingdou Shan, Sanxianchang, 1144 m, 17–19.V.2017, leg. Li Henan. **Jiangxi** (IZCAS): 1♂, Kuling, 13.VI.1974, leg. Zhang Baolin. **Hunan** (IZCAS): 1♂, Sangzhi, Bamaoxi, Shuitiannan, 370 m, 1.VIII.2009, leg. Wei Zhongmin; 1♂1♀, Sangzhi, Bamaoxi, Shuitianba, 370 m, 5–6.VIII.2009, leg. Wei Zhongmin; 1♂, Sangzhi Badagong Shan, Xiaozhuangping, 1420 m, 18.VI.2015, leg. Yao Jian, Zhao Kaidong; 2♂1♀, Yongshun, Xiaoxixiang, Xiaoxicun, 463–506 m, 21–24.IV.2018, leg. Zhao Kaidong. **Guangxi** (IZCAS): 1♀, Mao’er Shan, Jiuniuchang, 1100 m, 11.VII.1985, leg. Fang Chenglai; 1♂, Mao’er Shan, Jiuniutang, 1146 m, 16.VIII.2012, leg. Cheng Rui; 1♀, Mao’er Shan, Antangping, 1579 m, 17–18.VIII.2012, leg. Cheng Rui.

#### Distribution.

China (Shanxi, Henan, Shaanxi, Gansu, Anhui, Zhejiang, Hubei, Jiangxi, Hunan, Guangdong, Guangxi, Sichuan).

**Figures 173–178.**
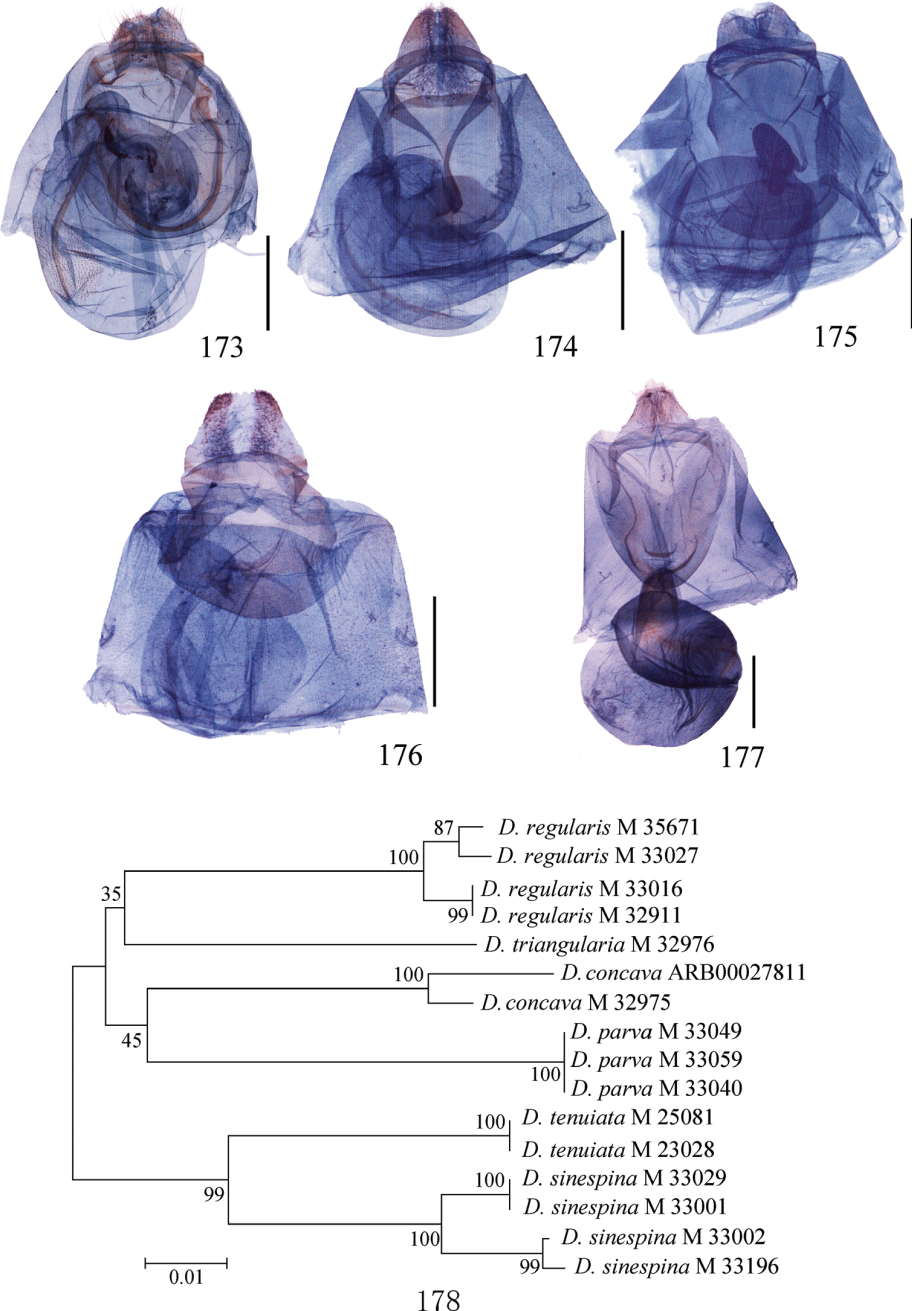
Female genitalia of *Ditrigona***173***D.berres***174***D.chama***175***D.platytes***176***D.clavata***177***D.cirruncata* Scale bars: 1 mm. **178** Neighbour-Joining (NJ) tree of selected *triangularia* species group based on the Kimura two-parameter model.

#### Remarks.

There are some variations in the eighth tergite (Figs 148–151), even in specimens collected from the same locality: for example, both Fig. 149 and Fig. 150 are from Emei Shan, Sichuan Province. Inoue (1962) recorded the distribution of *D.virgo* in central and west China, and was followed by Chu and Wang (1988, 1991). However, when checking the collection of IZCAS, only *D.cirruncata* was found. The record of *D.virgo* in China is doubtful. The situation is similar in *D.komarovi* (Kurentzov), a species recorded from Manchuria, and was combined from *Leucodrepana* by Wilkinson (1968). Chu and Wang (1988, 1991) recorded this species from Northeast China. However, when examining the collection of IZCAS, this species was not found, and its record in China is also doubtful.

## Supplementary Material

XML Treatment for Ditrigona

XML Treatment for Ditrigona derocina

XML Treatment for Ditrigona diana

XML Treatment for Ditrigona crystalla

XML Treatment for Ditrigona spilota

XML Treatment for Ditrigona inconspicua

XML Treatment for Ditrigona furvicosta

XML Treatment for Ditrigona jardanaria

XML Treatment for Ditrigona media

XML Treatment for Ditrigona innotata

XML Treatment for Ditrigona sericea

XML Treatment for Ditrigona pentesticha

XML Treatment for Ditrigona quinaria

XML Treatment for Ditrigona quinaria quinaria

XML Treatment for Ditrigona quinaria erminea

XML Treatment for Ditrigona quinaria spodia

XML Treatment for Ditrigona quinaria leucophaea

XML Treatment for Ditrigona obliquilinea

XML Treatment for Ditrigona obliquilinea thibetaria

XML Treatment for Ditrigona idaeoides

XML Treatment for Ditrigona triangularia

XML Treatment for Ditrigona uniuncusa

XML Treatment for Ditrigona tenuiata

XML Treatment for Ditrigona regularis

XML Treatment for Ditrigona sinespina

XML Treatment for Ditrigona parva

XML Treatment for Ditrigona concava

XML Treatment for Ditrigona titana

XML Treatment for Ditrigona sciara

XML Treatment for Ditrigona pomenaria

XML Treatment for Ditrigona polyobotaria

XML Treatment for Ditrigona typhodes

XML Treatment for Ditrigona lineata

XML Treatment for Ditrigona lineata lineata

XML Treatment for Ditrigona lineata tephroides

XML Treatment for Ditrigona legnichrysa

XML Treatment for Ditrigona policharia

XML Treatment for Ditrigona artema

XML Treatment for Ditrigona candida

XML Treatment for Ditrigona chionea

XML Treatment for Ditrigona fusca

XML Treatment for Ditrigona conflexaria

XML Treatment for Ditrigona conflexaria conflexaria

XML Treatment for Ditrigona conflexaria micronioides

XML Treatment for Ditrigona conflexaria cerodeta

XML Treatment for Ditrigona margarita

XML Treatment for Ditrigona berres

XML Treatment for Ditrigona chama

XML Treatment for Ditrigona platytes

XML Treatment for Ditrigona clavata

XML Treatment for Ditrigona marmorea

XML Treatment for Ditrigona quinquelineata

XML Treatment for Ditrigona aphya

XML Treatment for Ditrigona cirruncata
